# Golgi Protein 73 Promotes Angiogenesis in Hepatocellular Carcinoma

**DOI:** 10.34133/research.0425

**Published:** 2024-07-17

**Authors:** Yiming Liu, Xinyang Hu, Sining Zhou, Ting Sun, Feiyan Shen, Linghui Zeng

**Affiliations:** ^1^ Key Laboratory of Novel Targets and Drug Study for Neural Repair of Zhejiang Province, Hangzhou City University School of Medicine, Hangzhou 310015, China.; ^2^ Laboratory of Cancer Biology, Key Laboratory of Biotherapy of Zhejiang Province, Sir Run Run Shaw Hospital, Zhejiang University School of Medicine, Hangzhou 310017, China.; ^3^ Cancer Center, Zhejiang University, Hangzhou 310058, China.; ^4^ Life Sciences Institute, Zhejiang University, Hangzhou 310058, China.; ^5^ Department of Pathology, The First Affiliated Hospital, Zhejiang University School of Medicine, Hangzhou 310003, China.

## Abstract

Golgi protein 73 (GP73), a resident protein of the Golgi apparatus, is notably elevated in hepatocellular carcinoma (HCC). While its critical role in remodeling the tumor microenvironment (TME) is recognized, the intricate mechanisms are not fully understood. This study reveals that GP73 in HCC cells interacts with prolyl hydroxylase-2 (PHD-2) in a competitive manner, thereby impeding the hydroxylation of hypoxia-induced factor-1α (HIF-1α). The effect above promotes the production and secretion of vascular endothelial growth factor A (VEGFA). Moreover, exosomal GP73 derived from HCC cells can be internalized by human umbilical vein endothelial cells (HUVECs) and competitively interact with HECTD1, an E3 ubiquitin ligase targeting growth factor receptor-bound protein 2 (GRB2). This interaction stabilizes GRB2, thereby activating the Ras–mitogen-activated protein kinase (MAPK) signaling pathway. Consequently, escalated levels of GP73 intensify VEGF production in HCC cells and potentiate mitogenic signaling in vascular endothelial cells, fostering angiogenesis in the TME. Our findings propose that GP73 might serve as a novel target for anti-angiogenic therapy in HCC.

## Introduction

Hepatocellular carcinoma (HCC) ranks as the sixth most prevalent cancer and the third leading cause of cancer-related mortality globally [1]. Hepatitis B virus and hepatitis C virus infections are the predominant contributors to HCC, especially in China and other Asian regions [2,3]. Despite advances in HCC management, the disease poses significant challenges due to limited therapeutic options after recurrence and metastasis [4].

Angiogenesis, the process of new vessel formation, is crucial for cancer proliferation and spread [5]. Thus, impairing angiogenesis in the TME is a promising strategy for cancer management [6]. However, anti-angiogenesis therapies have not achieved comparable success in HCC as in other cancer types, reflecting the intricate nature of angiogenesis in HCC. Typically, the activation of vascular endothelial growth factor (VEGF)/VEGF receptor (VEGFR) signaling pathway in vascular endothelial cells drives angiogenesis within the TME, a process that can be reversed by blocking the VEGF-VEGFR interaction with specific antibodies and inhibitors, including bevacizumab and apatinib [7,8]. Nevertheless, combining these agents with other treatments, for example, immune checkpoint inhibitors (ICIs), could lead to severe side effects [9]. This necessitates the exploration of a novel therapeutic target activating both upstream and downstream of VEGF/VEGFR signaling cascade and other pivotal angiogenesis pathways.

Golgi protein 73 (GP73), a Golgi apparatus-associated protein encoded by *GOLM1*, is up-regulated in both tumor tissues and serum of HCC patients, functioning as a diagnostic biomarker [10,11]. Recent studies have highlighted the role of GP73 in remodeling the tumor immune microenvironment, thus hastening HCC advancement and metastasis [12,13]. Our preliminary observations suggest a positive correlation between GP73 expression and increased vascular density in tumor tissues. Given the established role of GP73 in transforming the TME, we hypothesize that GP73 may also promote angiogenesis within the TME. This study aims to decode the mechanisms by which GP73 influences angiogenesis within the TME and evaluate its potential as a target for anti-angiogenic therapy in HCC.

## Results

### GP73 facilitates angiogenesis in HCC

**Fig. 1.**
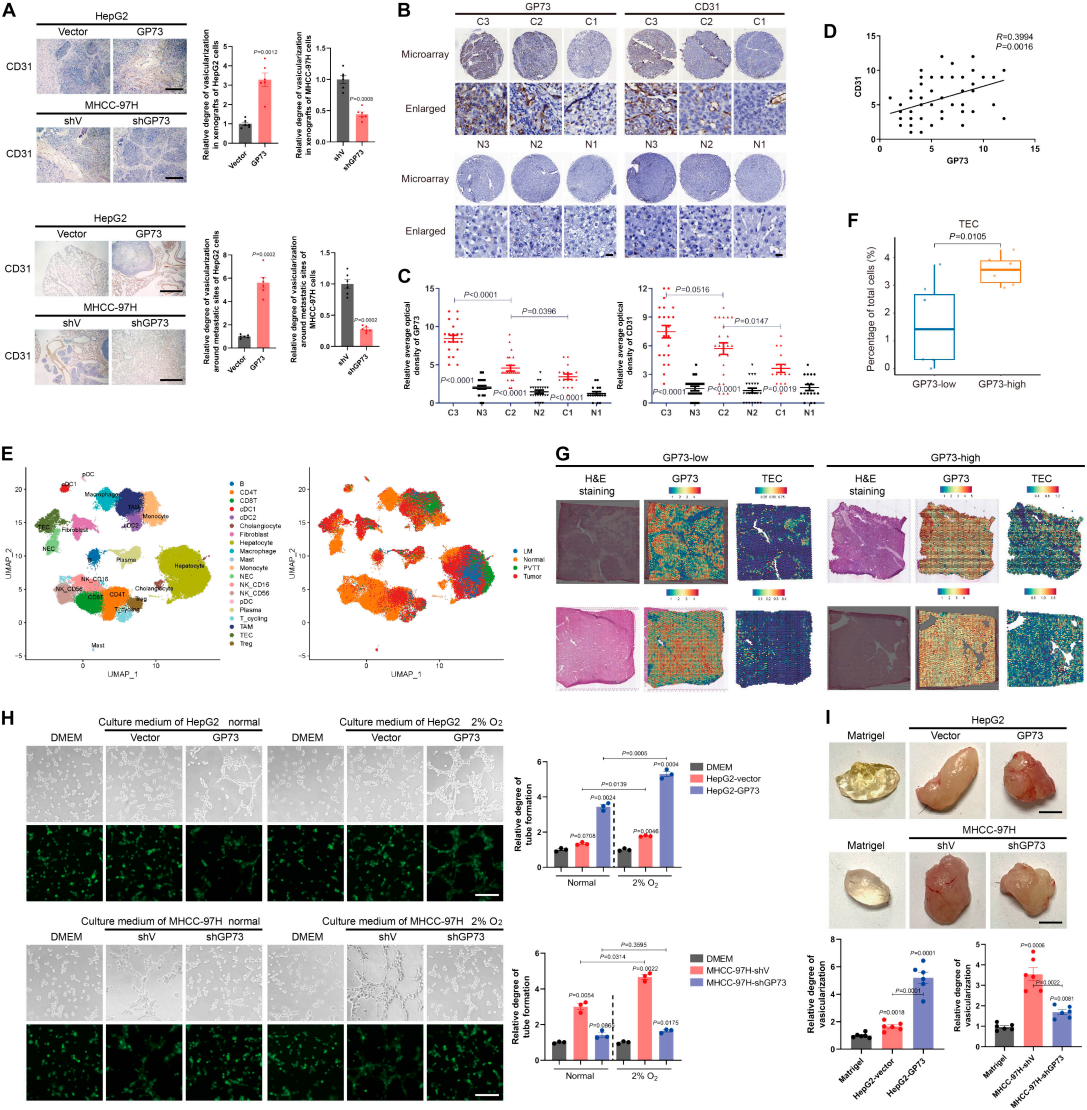
GP73 facilitates angiogenesis in HCC. (A) IHC staining of CD31 in xenograft (scale bar, 200 μm) and metastatic models (scale bar, 1 mm) of HepG2 and MHCC-97H cells (*n* = 6). (B) IHC analysis of GP73 and CD31 in primary tumor (*n* = 60) and adjacent liver (*n* = 60) tissues of HCC patients. Images were categorized by AJCC staging (I, II, and III; C, primary tumor; N, adjacent liver; scale bar, 10 μm). (C) The levels of GP73 and CD31 protein expression were shown as IHC scoring. (D) The correlation between the levels of GP73 and CD31 in primary tumor was analyzed using Spearman correlation analysis. (E) The Uniform Manifold Approximation and Projection (UMAP) plots showing the major cell types (left) and the distribution of the tissue types (right). NEC, normal endothelial cells; TAM, tumor-associated macrophage; TEC, tumor endothelial cells; LM, lymph node metastasis; PVTT, portal vein tumor thrombus. (F) Boxplots showing proportions of TEC between GP731-high and GP73-low tumors. (G) Hematoxylin and eosin (H&E) staining, GP73 spatial expression, and abundance of TEC are shown in GP731-high and GP73-low tumor samples from ST data. (H) HUVECs were treated with culture medium from indicated cells for 48 h, followed by a tube formation assay (scale bar, 50 μm; *n* = 3). (I) In vivo Matrigel plug implant model using C57/BL6 mice subcutaneously injected with Matrigel mixed with indicated cells. In vivo neovascularization was quantified using Darbkin’s reagent kit 7 d after injection (scale bar, 500 μm; *n* = 6). Data in (A), (C), (D), (F), (H), and (I) are presented as mean ± SEM. A 2-tailed Student’s *t* test was used for statistical analysis. Data in correlation analysis of (D) were analyzed using Spearman correlation analysis.

To investigate the influence of GP73 on angiogenesis, we performed immunohistochemical (IHC) staining for CD31, a vascular endothelial cell marker, on samples from in vivo tumor growth and metastasis models of HCC. Tumors with increased GP73 expression had a significantly higher vessel density compared to those with low GP73 expression (Fig. 1A), suggesting that GP73 may promote angiogenesis in HCC. Further IHC analyses of GP73 and CD31 were conducted on primary tumor tissues and corresponding adjacent liver tissues derived from HCC patients (*n* = 60; Fig. 1B). We observed an up-regulation of both GP73 and CD31 in primary tumors compared to adjacent liver tissues, with a positive correlation between their levels (*R* = 0.3994; Fig. 1C and D).

Next, to confirm the pro-angiogenic effect of GP73 in primary HCC, data of single-cell sequencing and spatial transcriptomic (ST) assays of primary tumor tissues derived from HCC patients were analyzed, categorizing them into GP73-high and GP73-low groups. The results of single-cell sequencing revealed that the proportion of tumor endothelial cells (TECs) in the GP73-high group was significantly greater than in the GP73-low group (Fig. 1E and F). Moreover, ST analysis showed that TECs are concentrated in primary HCC tissues with elevated GP73 levels and are predominantly located around tumor cells with up-regulated GP73 (Fig. 1G). Data above indicate that GP73 in HCC cells facilitates angiogenesis in the TME of HCC.

To determine if GP73 contributes to angiogenesis by influencing the release of angiogenic factors from HCC cells, culture medium from HepG2 and MHCC-97H cells with modified GP73 expression was cocultured with human umbilical vein endothelial cells (HUVECs). Results from cell proliferation and transwell migration assays indicated that culture medium from GP73 up-regulated HCC cells (after overexpression in HepG2 and before knockdown in MHCC-97H) facilitated the HUVEC proliferation and migration (Fig. S1A and B). Additionally, the conditioned medium from GP73 up-regulated HCC cells enhanced tube formation of HUVECs ex vivo, while the Matrigel plug assay confirmed the effects of HCC cell-derived GP73 on promoting angiogenesis in vivo (Fig. 1H and I). Notably, the pro-angiogenic effects were further amplified under hypoxic conditions, which are known to induce angiogenesis (Fig. S1A and B and Fig. 1H) [14]. Collectively, these findings indicate that GP73 originating from HCC cells promotes vascularization within the TME.

### GP73 stabilizes HIF-1α to facilitate VEGFA production in HCC cells

**Fig. 2.**
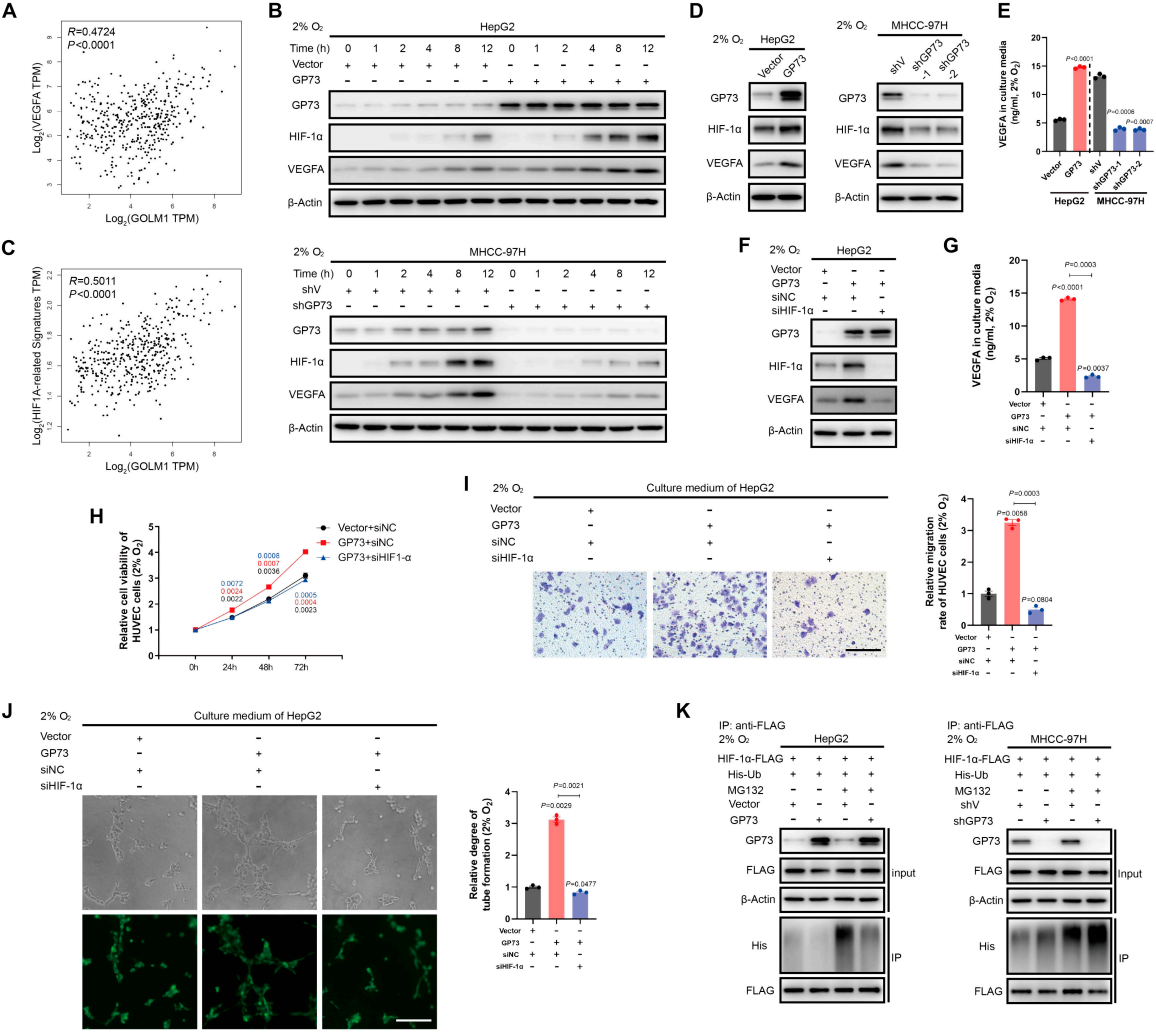
GP73 stabilizes HIF-1α to facilitate VEGFA production in HCC cells. (A) Gene Expression Profiling Interactive Analysis databases (GEPIA2) were utilized to analyze the correlations of mRNA levels of *GOLM1* and *VEGFA* in human liver hepatocellular carcinoma (LIHC). (B) Immunoblotting analysis of HIF-1α and VEGFA in indicated cells treated with 2% oxygen for 0, 1, 2, 4, 8, and 12 h. (C) Correlations of mRNA levels of *GOLM1*- and *HIF1A*-related signatures in human LIHC samples derived from GEPIA2 database. (D) Immunoblotting analysis of HIF-1α and VEGFA in HepG2 and MHCC-97H cells as indicated. (E) The content of VEGFA in cell culture medium from indicated cells was determined using a human VEGFA enzyme-linked immunosorbent assay (ELISA) kit 48 h after cell culture (*n* = 3). (F) Immunoblotting analysis of HIF-1α and VEGFA in HepG2 cells transfected with HIF-1α-specific siRNA for 48 h. (G) Content of VEGFA in cell culture medium from indicated cells transfected with HIF-1α-specific siRNA for 48 h (*n* = 3). (H) Cell viability was measured using MTS assay after HUVECs were treated with indicated cell culture medium for 0, 24, 48, and 72 h (*n* = 3). (I) Transwell cell migration assay of HUVECs (upper chamber) after coculturing with cell culture medium from indicated cells (lower chamber) for an additional 48 h (scale bar, 100 μm; *n* = 3). (J) Tube formation assay was performed after HUVECs were treated with indicated cell culture medium for 48 h (scale bar, 50 μm; *n* = 3). (K) The ubiquitination level of ectopic HIF-1α in indicated cells was determined by immunoblotting analysis 48 h after transfection. Cells were treated with MG132 (2 μM) 12 h before harvesting. Data in (A), (C), (E), and (G) to (J) are presented as mean ± SEM. A 2-tailed Student’s *t* test was used for statistical analysis. Data in correlation analysis of (A) and (C) were analyzed using Spearman correlation analysis.

Given the pivotal role of VEGFA in angiogenesis under hypoxia [15], it was supposed that GP73 could stimulate the production of VEGFA in HCC cells. Remarkably, the mRNA level of *VEGFA* positively correlated with that of *GOLM1* in primary tumor tissues derived from HCC patients (*R* = 0.4724; Fig. 2A). In addition, the levels of VEGFA and hypoxia-induced factor-1α (HIF-1α) were increased in GP73 overexpressed HepG2 cells and reduced in GP73 silenced MHCC-97H cells under hypoxia (Fig. 2B). It was revealed that once VEGFA was silenced in HCC cells, the conditioned medium from HCC cells could no longer promote the proliferation, migration, and tube formation of HUVEC (Fig. S2A to E), indicating that GP73-mediated angiogenesis is dependent on VEGFA up-regulation.

As VEGFA is known to be stimulated by up-regulated HIF-1α within the hypoxic TME [16], and it was shown that GP73 activated both HIF-1α and VEGFA under hypoxia, we considered that GP73 promotes VEGFA production via HIF-1α up-regulation. It was shown that the mRNA levels of *HIF1A*-related signatures were positively associated with that of *GOLM1* in primary tissues (*R* = 0.5011; Fig. 2C), and GP73 was found to up-regulate HIF-1α, thereby facilitating production and secretion of VEGFA (Fig. 2D and E). Besides, silencing HIF-1α reduced the production and secretion of VEGFA in HepG2 cells with GP73 overexpression (Fig. S2F and Fig. 2F and G) and significantly impaired GP73-induced proliferation, migration, and tube formation of HUVEC (Fig. 2H to J), It suggests that GP73 up-regulates VEGFA through HIF-1α to promote angiogenesis.

Given that GP73 had little impact on the mRNA level of *HIF1A* (Fig. S2G), GP73 appears to modulate the hypoxia-induced stabilization of HIF-1α rather than its synthesis. Further analysis indicated that GP73 inhibited the ubiquitination of both endogenous and ectopic HIF-1α to enhance its stability (Fig. S2H and Fig. 2K), supporting that GP73 underpins HIF-1α stabilization, which in turn elevates the production of VEGFA.

### GP73 interacts with PHD-2 to inhibit HIF-1α degradation in HCC cells

**Fig. 3.**
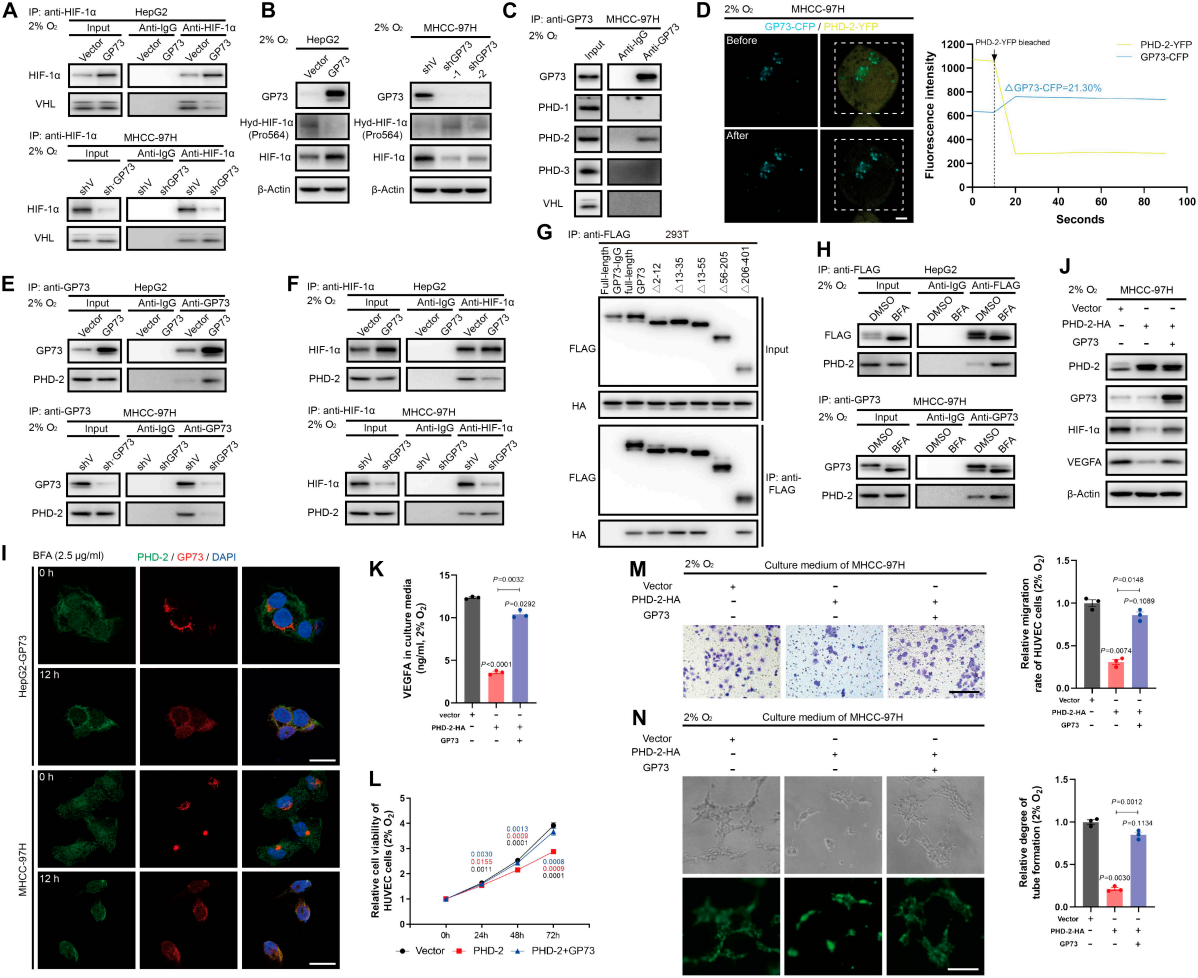
GP73 interacts with PHD-2 to inhibit HIF-1α degradation in HCC cells. (A) The interaction between HIF-1α and VHL in HepG2 and MHCC-97H cells was determined using co-IP followed by immunoblotting analysis. (B) The hydroxylation level of HIF-1α in indicated cells was determined by immunoblotting analysis. (C) The interaction between GP73 and HIF-1α degradation-related factors in MHCC-97H cells was determined using co-IP followed by immunoblotting analysis. (D) The direct interaction between GP73-CFP and PHD-2-YFP fusion proteins in MHCC-97H cells was determined by FRET analysis (scale bar, 2 μm). (E) The interaction between GP73 and PHD-2 in indicated cells was determined using co-IP followed by immunoblotting analysis. (F) The interaction between HIF-1α and PHD-2 in indicated cells was determined using co-IP followed by immunoblotting analysis. (G) Mapping of the binding site of GP73 and PHD-2. (H) The interaction between GP73 and PHD-2 in indicated cells was determined using co-IP followed by immunoblotting analysis. Cells were treated with BFA (2.5 μg/ml) for 0 and 12 h before harvesting. (I) Immunofluorescence staining of PHD-2 (green) and GP73 (red) after HepG2-GP73 and MHCC-97H cells were treated with BFA (2.5 μg/ml) for 0 and 12 h (scale bar, 10 μm). (J) Immunoblotting analysis of HIF-1α and VEGFA in MHCC-97H cells expressing ectopic GP73 or PHD-2 for 48 h. (K) Content of VEGFA in cell culture medium from MHCC-97H cells expressing ectopic GP73 or PHD-2 for 48 h (*n* = 3). (L) Cell viability was measured using MTS assay after HUVECs were treated with cell culture medium from MHCC-97H cells expressing ectopic GP73 or PHD-2 for 0, 24, 48, and 72 h (*n* = 3). (M) Transwell cell migration assay of HUVECs (upper chamber) cocultured with cell culture medium from indicated cells (lower chamber) for 48 h (scale bar, 100 μm; *n* = 3). (N) Tube formation assay was performed after HUVECs were treated with cell culture medium from MHCC-97H cells expressing ectopic GP73 or PHD-2 for 48 h (scale bar, 50 μm; *n* = 3). Data in (K) to (N) are presented as mean ± SEM. A 2-tailed Student’s *t* test was used for statistical analysis.

Contrary to our assumption that GP73 directly prevents HIF-1α degradation, co-immunoprecipitation (co-IP) followed by immunoblotting showed no interaction between HIF-1α and GP73 (Fig. S3A). This prompted us to explore whether GP73 modulated upstream regulators of HIF-1α to prolong its stabilization. However, it showed negligible effects of GP73 on prolyl hydroxylase-1/2/3 (PHD-1/2/3) and VHL, the regulators associated with HIF-1α degradation (Fig. S3B) [17]. Upon GP73 overexpression, an attenuated interaction between VHL and HIF-1α was noted (Fig. 3A). Given that hydroxylation at proline 564 of HIF-1α is essential for VHL-mediated degradation [18], we discovered that GP73 negatively regulates this hydroxylation process (Fig. 3B).

Subsequent co-IP followed by immunoblotting analysis established PHD-2 as an associate of GP73 (Fig. 3C). Fluorescence resonance energy transfer (FRET) assay demonstrated that the fluorescence intensity of cyan fluorescent protein (CFP) increased 21.30%, proving that GP73 directly interacts with PHD-2 under hypoxia (Fig. 3D). Additionally, the presence of GP73 diminished the interaction between PHD-2 and HIF-1α, suggesting a competitive inhibition of HIF-1α hydroxylation by GP73 (Fig. 3E and F). Mapping of the binding site of GP73 and PHD-2 indicated that GP73 interacted with PHD-2 through its intra-vesicular domain, implying that cytoplasmic GP73 might intercede in the process rather than Golgi-resident GP73 (Fig. 3G). Indeed, disrupting Golgi vesicle formation with brefeldin A (BFA) intensified the interaction and colocalization of GP73 and PHD-2 (Fig. 3H and I), thereby increasing HIF-1α levels in a cytoplasmic GP73-dependent pathway (Fig. S3C). Additional analyses demonstrated that the effect of PHD-2-induced HIF-1α degradation and VEGFA inhibition could be reversed by GP73 overexpression (Fig. 3J and K). Hence, without GP73 overexpression, PHD-2-rich HCC cell conditioned media inhibited HUVEC growth, migration, and vascularization compared to media from cells where both PHD-2 and GP73 were overexpressed (Fig. 3L to N). In conclusion, GP73 functions to stabilize HIF-1α by competitively interacting with PHD-2, thus activating the HIF-1α–VEGFA axis in HCC cells, which promotes angiogenesis within the hypoxic TME.

### HCC cell-derived exosomal GP73 promotes angiogenesis

Although the HIF-1α–VEGFA axis is renowned for driving angiogenesis under hypoxia [19], the conditioned medium from HCC cells with high GP73 expression could still stimulate the proliferation, migration, and tube formation of HUVECs under normoxic conditions (Fig. 1E to G). This suggests that GP73 may facilitate angiogenesis through alternative mechanisms. Recent studies have reported that GP73-enriched exosomes can contribute to cancer progression [12,13], leading to speculation about whether and how exosomes from HCC cells with up-regulated GP73 promote vascularization within the TME. Notably, the impacts on HUVEC proliferation, migration, and tube formation by the conditioned medium from HCC cells with GP73 up-regulation were significantly reduced upon exosome depletion (Fig. S4A to C), underscoring the potential role of GP73-enriched exosomes in angiogenic processes within the TME.

To evaluate the quality of exosomes derived from HCC cell culture medium, the diameter, structure, and potential contaminations of isolated exosomes were analyzed using nanoparticle tracking analysis and cryo-electron microscopy, which guaranteed that our samples were free from extracellular vesicle contamination (Fig. S5A and B). Furthermore, these exosomes were verified as free from subcellular fraction contamination by assessing exosomal and organelle-specific markers (Fig. S5C).

Further investigations revealed a positive correlation between the levels of exosomal GP73 isolated from HCC cell lines and their intracellular GP73 content (*R* = 0.8256; Fig. S5D). Abnormally expressed cytoplasmic GP73 resulted in its exosome-dependent secretion (Fig. S5E), which theoretically would regulate the concentration of GP73 in exosomes by modulating intracellular GP73.

**Fig. 4.**
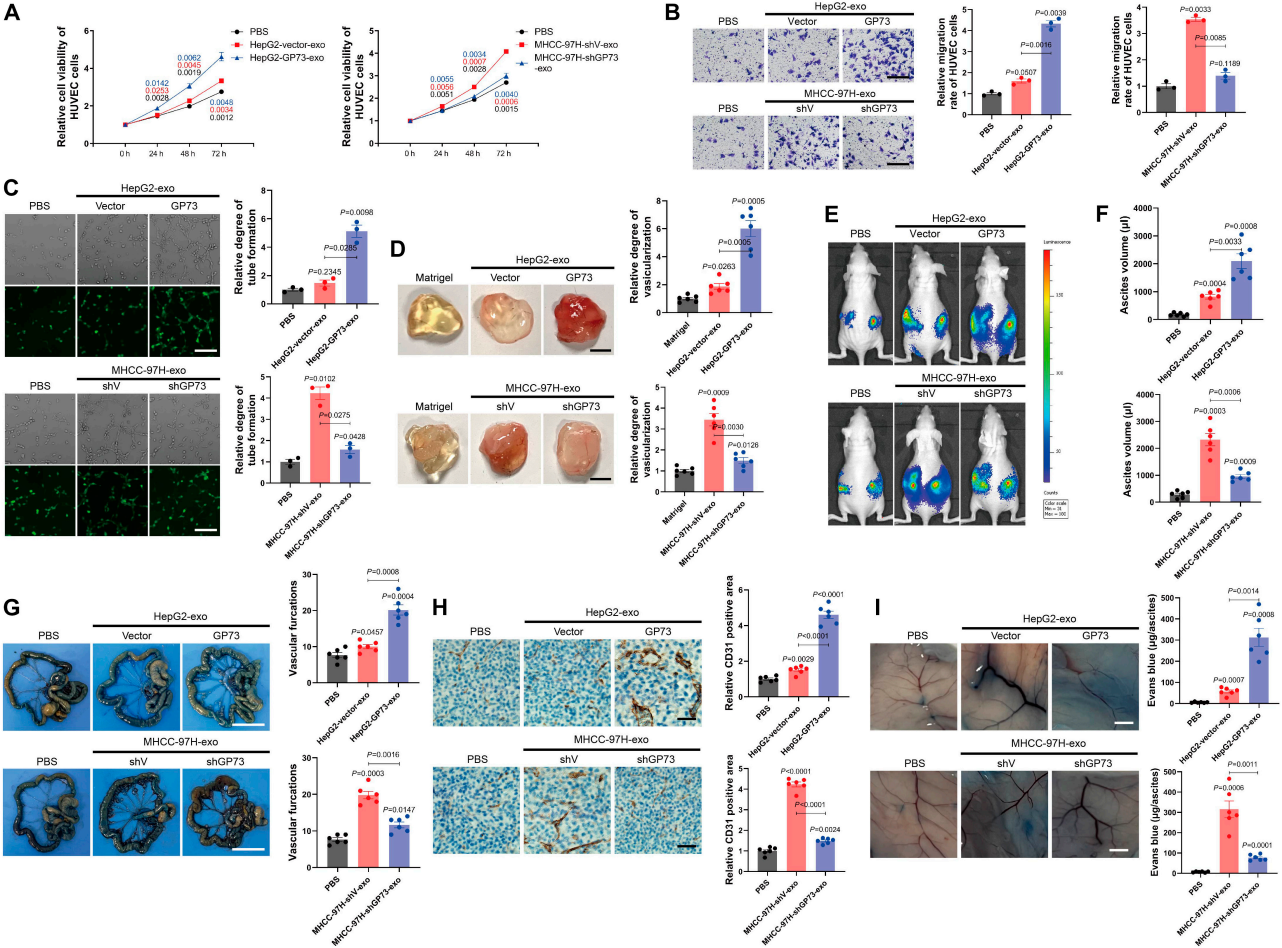
HCC cell-derived exosomal GP73 promotes angiogenesis. (A) MTS assay of HUVECs treated with exosomes derived from indicated cells for 0, 24, 48, and 72 h (*n* = 3). (B) Transwell cell migration assay of HUVECs (upper chamber) cultured with medium supplemented with exosomes derived from indicated cells for 48 h (scale bar, 100 μm; *n* = 3). (C) HUVECs were treated with exosomes derived from indicated cells for 48 h, followed by a tube formation assay (scale bar, 50 μm; *n* = 3). (D) In vivo Matrigel plug implant model using C57/BL6 mice subcutaneously injected with Matrigel mixed with exosomes derived from indicated cells. In vivo neovascularization was quantified using Darbkin’s reagent kit 7 d after injection (scale bar, 500 μm; *n* = 6). (E) Intraperitoneal dissemination assay using exosomes derived from indicated cells. Nude mice were treated intraperitoneally with indicated exosomes (30 μg per dose) every other day for 21 d. Metastatic sites were visualized by bioluminescence before sacrificing. (F) Metastatic extents were represented by the volume of ascitic fluids (*n* = 6). (G) Vascularization around mesentery was represented using vascular furcation (scale bar, 2 cm; *n* = 6). (H) IHC staining of CD31 in neoplasms around the mesentery (scale bar, 20 μm; *n* = 6). (I) Representative images of Evans blue extravasation in the abdominal wall (scale bar, 2.5 mm; *n* = 6). Evans blue extravasated into ascites was quantified spectrophotometrically at 595 nm. Data in (A) to (D) and (F) to (I) are presented as mean ± SEM. A 2-tailed Student’s *t* test was used for statistical analysis.

Crucially, exosomes from HCC cells with highly expressed GP73 promoted the proliferation, migration, tube formation, and in vivo angiogenesis of HUVECs or mouse vascular endothelial cells (Fig. 4A to D). Additionally, introducing these exosomes into mice inoculated intraperitoneally with HCC cells promoted tumor metastasis and induced more ascites (Fig. 4E and F), with a noticeable increase in vascularization around the mesentery (Fig. 4G). IHC analysis of CD31 and Evans blue dye extravasation further confirmed that exosomes from HCC cells with elevated GP73 expression augmented neovascularization within the TME (Fig. 4H and I). Taken together, the results above demonstrate the potent pro-angiogenic effects of exosomes derived from HCC cells with high GP73 levels both ex vivo and in vivo.

### Exosomes from GP73 up-regulated HCC cells enhance Ras activation by stabilizing GRB2

**Fig. 5.**
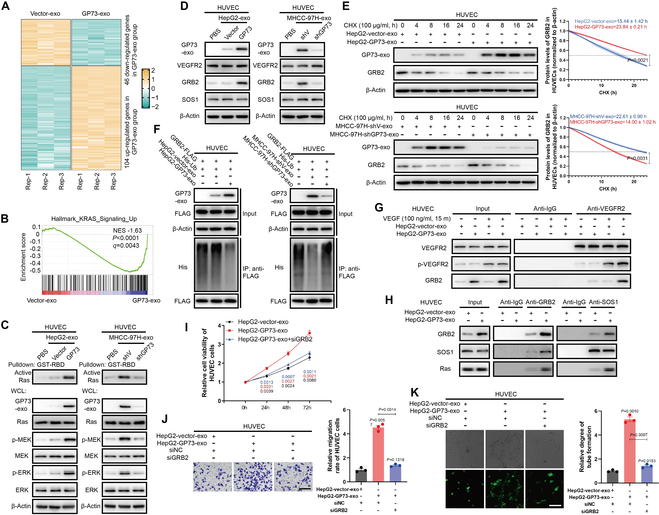
Exosomes from GP73 up-regulated HCC cells enhance Ras activation by stabilizing GRB2. (A) Heatmap of mRNA expression fold change in HUVECs treated with exosomes derived from HepG2 cells. (B) GSEA indicated that exosomes derived from GP73 up-regulated cells activated Ras-related signaling pathways. (C) RBD-pulldown and immunoblotting analysis of Ras-related factors in HUVECs treated with exosomes derived from indicated cells for 48 h. (D) Immunoblotting analysis of GRB2 and SOS1 in HUVECs treated with exosomes derived from indicated cells for 48 h. (E) The half-life of GRB2 in HUVECs treated with exosomes derived from HepG2 and MHCC-97H cells was determined by immunoblotting analysis (*n* = 3). (F) The ubiquitination level of GRB2-FLAG in HUVECs transfected with indicated plasmids and treated with exosomes derived from indicated cells was determined by immunoblotting analysis. (G) The interaction between VEGFR2 and GRB2 in HUVECs transfected with GRB2-specific siRNA and treated with exosomes derived from HepG2 cells was determined using co-IP followed by immunoblotting analysis. (H) The interaction between GRB2, SOS1, and Ras in HUVECs transfected with GRB2-specific siRNA and treated with exosomes derived from HepG2 cells was determined using co-IP followed by immunoblotting analysis. (I) MTS assay of HUVECs transfected with GRB2-specific siRNA for 48 h and treated with exosomes derived from indicated cells for 0, 24, 48, and 72 h (*n* = 3). (J) Transwell cell migration assay of HUVECs transfected with GRB2-specific siRNA for 48 h and cocultured with exosomes (added to cell culture medium in upper chamber) derived from indicated cells for 48 h (scale bar, 100 μm; *n* = 3). (K) HUVECs were transfected with GRB2-specific siRNA and treated with exosomes derived from indicated cells for 48 h, followed by a tube formation assay (scale bar, 50 μm; *n* = 3). Data in (A) were analyzed using nonparametric test. Data in (B) were analyzed using permutation test. Data in (E) and (I) to (K) are presented as mean ± SEM. A 2-tailed Student’s *t* test was used for statistical analysis.

To elucidate the mechanism by which GP73-enriched exosomes from HCC cells promote angiogenesis, we profiled the total RNA expressions of HUVECs treated with exosomes from HepG2-V and HepG2-GP73 cells (Fig. S6A and B and Fig. 5A). Gene set enrichment analysis (GSEA) demonstrated that K-Ras-related signatures were significantly up-regulated in HUVECs treated with exosomes derived from HepG2-GP73 cells (Fig. 5B). The receptor-binding domain (RBD)-pulldown assay corroborated increased Ras activation in HUVECs exposed to exosomes from cells with GP73 up-regulation, which subsequently activated the mitogen-activated protein kinase (MAPK) kinase (MEK) and extracellular signal–regulated kinase 1/2 (ERK1/2) pathways (Fig. 5C).

We then examined receptor tyrosine kinase (RTK)-binding protein levels to decipher the mechanism behind Ras activation in HUVECs by exosomes from HCC cells with highly expressed GP73. The expression levels of VEGFR and SOS1 remained unchanged; however, the level of growth factor receptor-bound protein 2 (GRB2) was significantly increased in HUVECs treated with exosomes from GP73 up-regulated HCC cells (Fig. 5D). Curiously, the GRB2 mRNA levels in HUVECs were largely unaffected after exosome treatment (Fig. S6C). Yet, the protein stability of GRB2 was noticeably enhanced in HUVECs upon treatment with exosomes from HCC cells with highly expressed GP73 (Fig. 5E), along with a decrease in polyubiquitination of GRB2 (Fig. 5F), suggesting that exosomes from GP73 up-regulated HCC cells may stabilize GRB2 by impeding its ubiquitin–proteasome degradation pathway.

Subsequent to recombinant VEGF treatment, the interaction between VEGFR and GRB2, as well as the assembly of the GRB2/SOS1/Ras complex, was augmented in HUVECs treated with exosomes from HepG2-GP73 cells (Fig. 5G and H). Importantly, the angiogenic effects of exosomes from HCC cells with high GP73 expression on HUVEC were markedly weakened when GRB2 was silenced using GRB2-specific small interfering RNA (siRNA) (Fig. 5I to K). Thus, exosomes from HCC cells with increased GP73 expression potentiate Ras activation, facilitating angiogenesis by promoting GRB2 stability in HUVECs.

### Exosomal GP73 up-regulates GRB2 to activate Ras/MAPK signaling in HUVECs

**Fig. 6.**
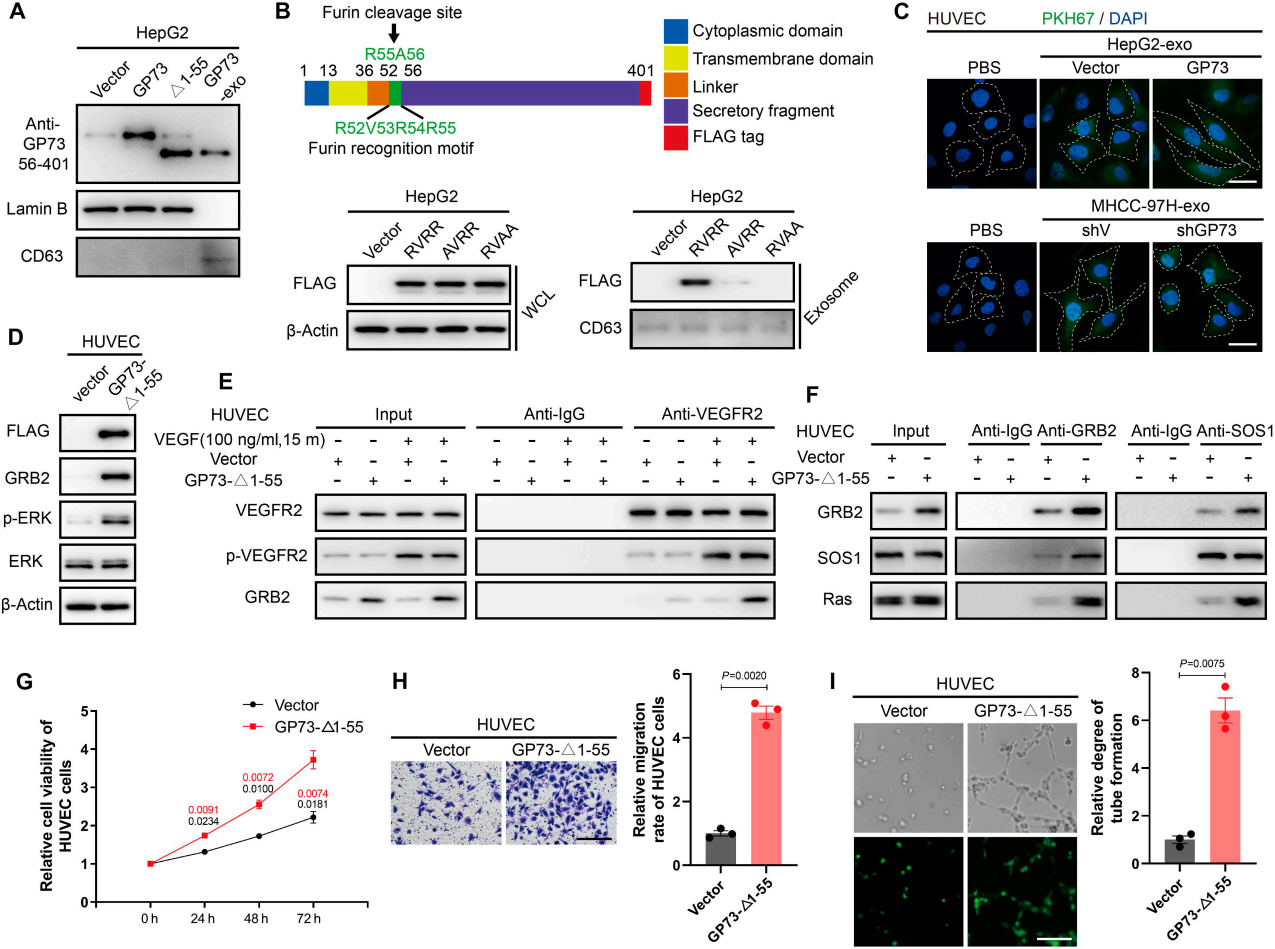
Exosomal GP73 up-regulates GRB2 to activate Ras/MAPK signaling in HUVECs. (A) Immunoblotting analysis of GP73 in HepG2 cells overexpressing full-length GP73 or △1-55 mutant or treated with exosomes derived from HepG2-GP73 cells for 48 h. (B) Immunoblotting analysis of intracellular and exosomal GP73 in HepG2 cells overexpressing GP73 or its RVRR domain mutants for 48 h. (C) HUVECs were treated with PKH67 (green)-labeled exosomes (working concentration: 20 μg/ml) derived from HepG2 and MHCC-97H cells (scale bar, 10 μm). (D) Immunoblotting analysis of GRB2 and p-ERK in HUVECs stably expressing vector and GP73-△1-55 mutant. (E) The interaction between VEGFR2 and GRB2 in indicated HUVECs was determined using co-IP followed by immunoblotting analysis. (F) The interaction between GRB2, SOS1, and Ras in indicated HUVECs was determined using co-IP followed by immunoblotting analysis. (G) MTS assay of indicated HUVECs cultured for 0, 24, 48, and 72 h (*n* = 3). (H) Transwell cell migration assay of indicated HUVECs cultured for 48 h (scale bar, 100 μm; *n* = 3). (I) Tube formation assay of indicated HUVECs cultured on Matrigel for 8 h (scale bar, 50 μm; *n* = 3). Data in (G) to (I) are presented as mean ± SEM. A 2-tailed Student’s *t* test was used for statistical analysis.

Consistent with previous findings [13], GP73 has been detected in exosomes originating from HCC cells exhibiting differential GP73 expression (Fig. S5D). Intriguingly, we discovered that the molecular weight of exosomal GP73 is less than that of the full-length GP73, mirroring a truncated variant lacking the initial 55 amino acids (Fig. 6A). Given that furin cleaves GP73 at the R55 position, facilitating its release from HCC cells [20], we engineered mutations within the furin recognition site of GP73. We found that substituting R52 or both R54 and R55 with alanine obstructed GP73 incorporation into exosomes, establishing the critical nature of the first 55 amino acids for GP73 packaging into exosomes (Fig. 6B).

With the internalization capacity of exosomes derived from HCC cells by HUVECs (Fig. 6C), we hypothesized that exosomal GP73, originating from HCC cells, might exert functional effects within HUVECs. Indeed, the ectopic expression of GP73 deprived of the initial 55 amino acids (GP73-Δ1-55) in HUVECs led to an upsurge in GRB2 protein levels (Fig. 6D). Although VEGFR phosphorylation was minimally impacted by the GP73-Δ1-55 variant, the interaction between VEGFR and GRB2 was notably enhanced, even without recombinant VEGF stimulation (Fig. 6E). Consequently, the presence of the GP73-Δ1-55 variant supported the formation of the GRB2/SOS1/Ras complex (Fig. 6F), leading to the effective activation of ERK1/2 (Fig. 6D). Thus, the proliferation, migration, and tube formation of HUVECs were significantly encouraged by GP73-Δ1-55 variant (Fig. 6G to I). These data indicate that exosomal GP73 from HCC cells elevates GRB2 levels to ignite Ras/MAPK signaling in HUVECs.

### Exosomal GP73 interacts with HECTD1 to inhibit GRB2 degradation in HUVECs

**Fig. 7.**
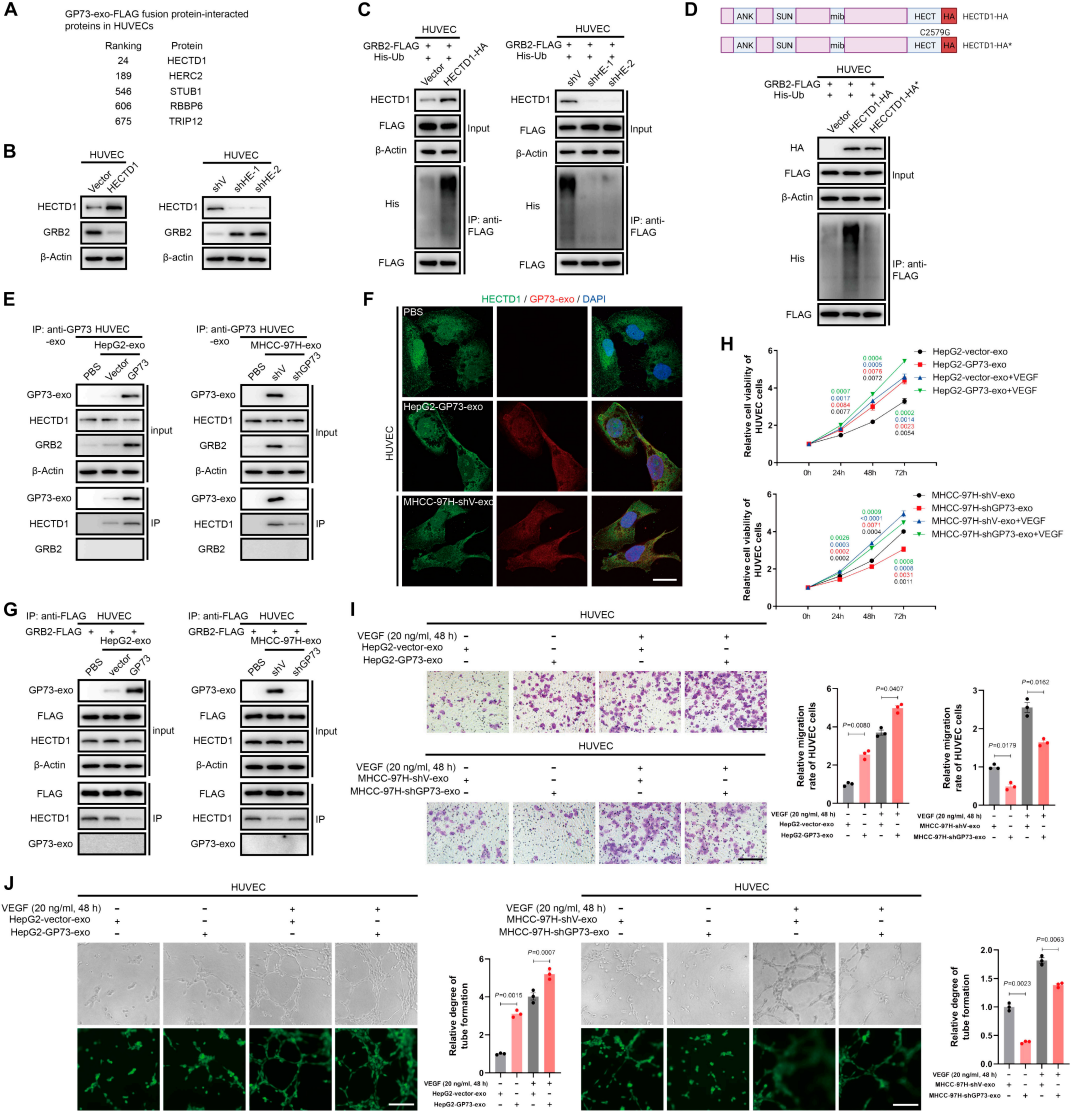
Exosomal GP73 interacts with HECTD1 to inhibit GRB2 degradation in HUVECs. (A) Identification of E3 ubiquitin ligases interacting with exosomal GP73 in HUVECs using liquid chromatography–tandem mass spectrometry (LC-MS/MS). (B). Immunoblotting analysis of GRB2 in HUVECs transfected with indicated plasmids and siRNAs for 48 h. (C) The ubiquitination level of GRB2-FLAG in HUVECs 48 h after transfection with indicated plasmids and siRNAs was determined by immunoblotting analysis. (D) The ubiquitination level of GRB2-FLAG in HUVECs 48 h after transfection with indicated plasmids was determined by immunoblotting analysis. (E) The interaction between exosomal GP73 and HECTD1 in HUVECs treated with exosomes for 48 h was determined using co-IP followed by immunoblotting analysis. (F) Immunofluorescence staining of HECTD1 (green) and exosomal GP73 (red) after HUVECs were treated with exosomes derived from GP73 up-regulated cells for 48 h (scale bar, 10 μm). (G) The interaction between GRB2 and HECTD1 in HUVECs treated with exosomes for 48 h was determined using co-IP followed by immunoblotting analysis. (H) MTS assay of HUVECs treated with exosomes derived from indicated cells for 0, 24, 48, and 72 h (*n* = 3). (I) Transwell cell migration assay of HUVECs (upper chamber) after coculturing with exosomes (added to cell culture medium in upper chamber) derived from indicated cells for 48 h (scale bar, 100 μm; *n* = 3). (J) HUVECs were treated with exosomes derived from indicated cells for 48 h, followed by a tube formation assay (scale bar, 50 μm; *n* = 3). Data in (H) to (J) are presented as mean ± SEM. A 2-tailed Student’s *t* test was used for statistical analysis.

To comprehend how exosomal GP73 up-regulates GRB2 in HUVECs, the interaction partners of exosomal GP73 within HUVECs after treatment with exosomes derived from HCC cells were identified using mass spectrometry. HECTD1, among several E3 ubiquitin ligases, was pinpointed as a partner interacting with exosomal GP73 (Fig. 7A). The selective silencing of HECTD1, as opposed to other E3 ligases, caused an up-regulation of GRB2 by undermining its polyubiquitination (Fig. S7A and B), hinting at a possible role for HECTD1 as a GRB2-targeting E3 ubiquitin ligase. Subsequent studies indicated that HECTD1 negatively regulated GRB2 (Fig. 7B), and congruently, the overexpression of HECTD1 intensified while its suppression dimmed the polyubiquitination levels of GRB2 (Fig. 7C). In addition, a HECTD1 mutant (HECTD1-C2579G), which is defective in ligase activity [21], failed to ubiquitinate GRB2, corroborating the direct targeting role of HECTD1 on GRB2 (Fig. 7D).

Upon the incorporation of HCC cell-derived exosomes into the HUVEC culture medium, exosomal GP73 was internalized and interacted with HECTD1 (Fig. 7E and F), thereby diminishing the binding between HECTD1 and GRB2 within HUVECs (Fig. 7G). However, exosomes from HCC cells with low GP73 expression no longer disturbed HECTD1-GRB2 interactions. This implies that exosomal GP73 competitively engages with HECTD1, impeding its interaction with GRB2 within HUVECs. Notably, recombinant VEGF effectively activated VEGFR/MAPK signaling, promoting the proliferation, migration, and tube formation of HUVECs, and the effects were further amplified by the addition of exosomes derived from HCC cells with elevated GP73 expression (Fig. S7C and Fig. 7H to J). Therefore, up-regulated GP73 in HCC cells contributes to the maximal activation of Ras/MAPK signaling, essential for effective angiogenesis, through the increased production of VEGF in HCC cells and the stabilization of GRB2 in vascular endothelial cells.

## Discussion

Recurrence and metastasis are the primary contributors to HCC-related mortality [4]. The copious vascularization of liver affords a nutrient-rich environment that nurtures tumor growth [22]. Moreover, the vascular network within the TME creates pathways for HCC cell migration, thereby promoting metastatic spread [23]. Consequently, understanding vascularization within the TME of HCC is critical for addressing cancer progression and has sparked intensive research into angiogenesis inhibitors. Recent advances have seen the successful application of monoclonal antibodies and kinase inhibitors directed against VEGFA and its receptors (VEGFR1/2/3) in combating non-small cell lung cancer (NSCLC), HCC, and other malignancies [8,9,24]. Despite their efficacy, these agents can elicit adverse reactions, including vascular disruptions and vessel regression, leading to drug resistance [25]. Although drug resistance may be mitigated by combining antibodies and inhibitors against VEGFA and VRGFR1/2/3, the side effects may be more severe than they are used separately [26]. Therefore, a modulator that concurrently targets the VEGFA and VEGFR1/2/3 signaling pathways might represent a more desirable therapeutic strategy for inhibiting angiogenesis.

In this study, we have discovered that GP73 promotes angiogenesis through 2 synergistic mechanisms: enhancing VEGFA secretion from HCC cells and amplifying VEGFR/MAPK signaling in endothelial cells. GP73 elevates VEGFA levels by stabilizing HIF-1α, a transcription factor that activates VEGFA expression under low oxygen conditions. Additionally, exosomal GP73 released by HCC cells binds to HECTD1 in vascular endothelial cells, competitively inhibiting its mediation of GRB2 degradation and consequently potentiating MAPK signaling. Thus, therapeutically targeting GP73 could impede both VEGFA production and VEGFR activation, signifying a promising avenue for intervention.

Given that GP73 is regarded as a biomarker for HCC diagnosis [27,28], and its rare expression in healthy tissue [29], it emerges as an optimal therapeutic target with minimal toxicity to normal cells. Since inhibition of GP73, the molecule that activates both VEGFA and VEGFR1/2/3 pathways, could effectively facilitate angiogenesis and decrease the risk of resistance induced by VEGF and VEGFR inhibitors, the pursuit of GP73-specific inhibitors mounts as a critical challenge in anti-angiogenesis therapy tailored to the TME of HCC. Furthermore, comparing the efficacy of a GP73 inhibitor with that of VEGFA/VEGFR1/2/3 inhibitors, alone or in combination with other therapies such as ICIs, presents an exciting research prospect.

This study also highlights a strong association between GP73 and CD31 in HCC primary tumors, positing serum GP73 as a potential marker evaluating TME vascularization.

Notably, our study discovered the unconventional roles of cytoplasmic GP73, in contrast with Golgi-associated GP73. Cytoplasmic GP73 could serve as an adaptor competitively inhibiting the activity of PHD-2 and HECTD1, resulting in the maximal activation of RTK-MAPK signaling pathways in vascular endothelial cells. The exact mechanisms by which cytoplasmic GP73 interacts with its targets remain elusive, posing an intriguing avenue for future inquiry, especially in its posttranslational modifications, such as glycosylation, under various physiological conditions [30].

In summary, the present work elucidates the significant role of GP73 from HCC cells in promoting angiogenesis within the TME. Considering the dual impact of both cytoplasmic and exosomal forms of GP73 on the VEGFR2 signaling axis, investigating inhibitors that target GP73 represents a promising strategy to counteract angiogenesis within the TME of HCC.

## Materials and Methods

### Cell culture

MHCC-97H cells were from the Liver Cancer Institute (Zhongshan Hospital, Fudan University, China); SK-Hep-1, HepG2, Hep3B, PLC, and 293T cells were from the American Type Culture Collection (ATCC; Manassas, VA, USA); L02, HepG2.2.15, and Huh-7 cells were from National Collection of Authenticated Cell Cultures (NCACC; Shanghai, China). Cells were cultured in RPMI 1640 (L02 cells) or Dulbecco’s modified Eagle’s medium (DMEM) (other cell lines) (Thermo Fisher Scientific, Carlsbad, CA, USA) supplemented with 10% fetal bovine serum (FBS; Thermo Fisher Scientific) in 5% CO_2_ at 37 °C. Hypoxia of the experiments labeled with 2% O_2_ was induced by placing cells in a Forma 3 incubator (4110, Thermo Fisher Scientific) containing 2% O_2_. Cell lines above were authenticated by STR profiling at Cobioer Bioscience Co. Ltd. (Nanjing, China), and experiments were performed within <10 passages after authentication.

### Collection and treatment of clinical specimens

HCC pathological tissues and corresponding adjacent liver tissues derived from HCC patients were from Sir Run Run Shaw Hospital, Zhejiang University School of Medicine (Hangzhou, China). HCC patients were classified as the 8th edition staging system of the American Joint Committee on Cancer (AJCC) for HCC. Tissues were embedded with paraffin and manufactured into tissue microarrays for IHC analysis as previously described [31]. All samples were collected with the informed consent of the patients, and the experiments were approved by the Research Ethics Committee of Sir Run Run Shaw Hospital. Details of information of HCC patients are shown in Table S1.

### Analysis of the single-cell RNA-sequencing and ST data

The HCC single-cell RNA-sequencing (RNA-seq) data were obtained from the GSE149614 dataset. Data preprocessing was performed using the “Scanpy” Python software (V 1.9.8). Data integration and batch effect removal were carried out using the “scvi-tools” Python software (V 1.1.2). Dimensionality reduction was performed using the “tl.umap” function in the “Scanpy” Python software, and clustering was conducted using the “tl.leiden” function. Finally, cell type annotation was performed based on previous literature and classical marker genes. The HCC ST data were obtained from the study by Chen and colleagues [32]. The “Tangram” Python software (V 1.0.4) was used to predict the gene spatial expression of the ST data. The “cell2location” Python software (V 0.1.3) was utilized for deconvolution of the ST data to assess the abundance of different cell types.

### Tube formation assay

Growth factor-reduced Matrigel (356231, Corning, NY, USA) was tiled onto 24-well plate (250 μl/well) and incubated in 5% CO_2_ at 37 °C for 30 min. Culture medium or exosomes derived from indicated HCC cells were mixed with 75,000 HUVEC-GFP (green fluorescent protein) cells and incubated in 5% CO_2_ at 37 °C for extra 8 h. The remaining procedures were performed as previously reported [33]. Images were captured using an Olympus DP70 microscope in the bright field and 488-nm excitation light (Olympus Corporation, Tokyo, Japan).

### In vivo Matrigel plug assay

Growth factor-reduced Matrigel mixed with HCC cells (5 × 10^6^ cells) or exosomes derived from HCC cells (30 μg) were injected subcutaneously into 8-week-old C57/BL6 mice. Mice were sacrificed, and Matrigels were excised 7 d after injection. Degrees of vascularization were judged according to vessels on the surface of Matrigels. The new vessels were quantified using Whole Blood Hemoglobin (Hb) Kit (MAK455, Sigma-Aldrich, St. Louis, MO, USA) according to the manufacturer’s instructions.

### Immunoblotting analysis

Cells were lysed using radioimmunoprecipitation assay (RIPA) lysis buffer (20-188, Merck-Millipore, Billerica, MA, USA) with a protease and phosphatase inhibitor cocktail (78440, Thermo Fisher Scientific). Concentrations of lysates were determined using a BCA protein quantification kit (P0011, Beyotime, Nanjing, China), and samples were subjected to immunoblotting as previously described [34]. Antibodies used in immunoblotting analysis were shown in Table S2.

### Co-IP analysis

Cells were lysed using 1× IP lysis buffer (87787, Thermo Fisher Scientific) with a protease and phosphatase inhibitor cocktail for 30 min and centrifuged at 15,000*g* for 15 min. The supernatants were collected and incubated with the mixture of antibodies and protein A/G magnetic beads (88802, Thermo Fisher Scientific) for 6 h at 4 °C with gentle shaking. Proteins were eluted by IP elution buffer (0.1 M glycine, pH 2.0). For purification of exogenous proteins with FLAG-tag or HA-tag, proteins were co-immunoprecipitated using affinity gels shown in Table S2 and antigens were competitively eluted using DYKDDDDK Tag Synthetic Peptide (PEP087, Thermo Fisher Scientific) or HA synthetic peptide (11666975001, Merck-Millipore). Target proteins and their interacting proteins were examined using immunoblotting analysis or mass spectrometry.

### FRET analysis

MHCC-97H cells were cotransfected with pcDNA3-GP73-CFP and pcDNA3-PHD-2-YFP vectors and cultured for 48 h. Cells were cultured with 2% O_2_ for 12 h before FRET assay. FRET assay was performed with an FV3000 confocal microscope (Olympus Corporation, Tokyo, Japan).

The acceptor (PHD-2-YFP) was bleached using the 514-nm laser. The whole cell was imaged for both donor (GP73-CFP) and acceptor immediately before and after bleaching. FRET efficiency was calculated from the increase of the donor fluorescence intensity.

### Extraction and characterization of HCC cell-derived exosomes

HepG2 and MHCC-97H cells stably expressing GP73 or GP73-specific short hairpin RNAs were cultured using DMEM supplemented with 10% exosome-depleted FBS (EXO-FBS-250A-1, System Biosciences, Palo Alto, CA, USA) in 5% CO_2_ at 37 °C. Cell culture medium was centrifuged at 2,000*g* for 5 min and 10,000*g* for 30 min to remove debris. Supernatants were concentrated by ultrafiltration using an Amicon Ultra-15 Centrifugal Filter Unit (UFC9100, Merck-Millipore). Phosphate-buffered saline (PBS) was used as the exchange buffer. Exosomes in concentrated components were extracted using an ExoQuick-ULTRA EV Isolation Kit (EQULTRA-20A-1, System Biosciences) following the manufacturer’s instructions. Extracted exosomes were characterized using nanoparticle tracking analysis and electron microscopy as previously reported [35].

### Labeling and treatment of HCC cell-derived exosomes

To examine the internalization efficiency of HCC cell-derived exosomes by HUVECs ex vivo, exosomes were labeled using a PKH67 green fluorescent labeling kit (PKH67GL, Sigma-Aldrich) as previously reported. Labeled exosomes were purified using an exoEasy Maxi kit (76064, QIAGEN, Hilden, Germany). Purified exosomes were incubated with HUVECs (working concentration: 20 μg/ml) at 37 °C for 48 h. The exosome-treated cells were then fixed and stained with 4′,6-diamidino-2-phenylindole (DAPI) (D3571, Thermo Fisher Scientific). The fluorescence of PKH67 (488 nm) was examined using confocal microscopy with an FV3000 confocal microscope.

### Intraperitoneal dissemination assay

Intraperitoneal dissemination assay was performed as previously reported [36]. PLC cells stably expressing luciferase were intraperitoneally injected into nude mice (2 × 10^5^ cells per mouse), and tumor-bearing mice were treated intraperitoneally with indicated HepG2 and MHCC-97H cell-derived exosomes (30 μg per dose) every other day for 21 d. Mice were anesthetized with pentobarbital sodium, and metastatic sites were imaged using IVIS Lumina II Animal Imaging System (PerkinElmer, Waltham, MA, USA). Mice were then intravenously injected with Evans blue (E2129, Sigma-Aldrich, 80 mg/kg) and circulated for 40 min. At the time of sacrifice, ascites was collected and determined with a pipette. Colorectums were removed and vessels on mesentery were counted, and then the tissues were fixed with formalin and neovascularization was examined by IHC staining with antibodies against mouse CD31 (details in Table S2). Last, to measure vascular extravasation of Evans blue dye, abdominal walls were excised for imaging, and then Evans blue dye was extracted from ascites and the concentration was quantified spectrophotometrically at 595 nm.

### RNA-seq analysis

Purified exosomes from HepG2 cells stably expressing GP73 and vector control HepG2 cells were incubated with HUVECs for 24 h. Total RNA of HUVECs was extracted using MiniBEST Universal RNA Extraction Kit (TaKaRa, Dalian, China) following the manufacturer’s instructions. RNA was evaluated using Agilent 2100 Bioanalyzer (Agilent Technologies, Santa Clara, CA, USA). The libraries were constructed using TruSeq Stranded mRNA LTSample Prep Kit (Illumina, San Diego, CA, USA) following the manufacturer’s instructions. RNA-seq was performed on the Illumina sequencing platform (HiSeq X Ten). Fold change >2 or <0.5 and *P* < 0.05 were set as the threshold for significant differential expression. Heatmap, Gene Ontology (GO) enrichment, and Kyoto Encyclopedia for Genes and Genomes (KEGG) enrichment analysis were performed using R package. GSEA was performed using GSEA 4.1.0, and cell growth-related signaling pathways were analyzed.

### Identification of exosomal GP73 binding E3 ubiquitin ligase

FLAG and GP73-c-FLAG fusion proteins were expressed in HepG2 cells with pCMV-c-FLAG and pCMV-GP73-c-FLAG vectors, and exosomes were isolated 48 h after transfection. HUVECs were treated with exosomes above for 24 h. Besides, another group of HUVECs was transfected with pCMV-GP73-c-FLAG vector for 48 h. Cells were lysed using 1× IP lysis buffer, and co-IP analysis was performed with anti-FLAG M2 Gel (details in Table S2) as described above. Proteins were separated by SDS–polyacrylamide gel electrophoresis (PAGE), and target fragments were cropped and digested. Peptide fragments were lyophilized and suspended in 0.05% trifluoroacetic acid (TFA) solution. Then, they were separated and identified using Easy-nLC 1200 (Thermo Fisher Scientific) and Q Exactive (Thermo Fisher Scientific). Data were searched based on UniProt database (Human). Proteins were filtered as unused ≥1.3, peptide probability >95.0%, and protein probability >99.0%. E3 ubiquitin ligases that interacted with exosomal GP73 instead of intracellular GP73 were selected.

### Statistical analysis

Data were analyzed using a 2-tailed Student’s *t* test and shown as the mean ± SEM with the software Statistical Package for the Social Sciences (SPSS) version 20. Correlation analysis was computed by Spearman correlation analysis. *P* values < 0.05 were considered significant.

## Data Availability

All data in our study that support the findings are available from the corresponding authors upon reasonable request.

## Supplementary Materials

Supplementary 1 Materials and Methods Figs. S1 to S7 Tables S1 to S4 References
