# Supplementary material for: Golgi Protein 73 Promotes Angiogenesis in Hepatocellular Carcinoma
Source: Research (Wash D C). 2024 Jul 17;7:0425. doi: 10.34133/research.0425 (PMC11251733; doi:10.34133/research.0425)
Supplement: Supplementary 1 — Materials and Methods Figs. S1 to S7 Tables S1 to S4 References [file research.0425.f1.docx]

**Supplementary information**

**Supplementary materials and methods**

**Reagents**

All chemicals, including Brefeldin A (BFA, HY-16592), chloroquine (CQ, HY-17589A), Cycloheximide (CHX, HY-12320), MG132 (HY-13259), were purchased from MedChemExpress (Monmouth Junction, NJ, USA). Vascular endothelial growth factor (VEGF, 100-20) was purchased from PeproTech Inc. (Rocky Hill, NJ, USA).

**Plasmid and siRNA transfection**

The plasmids pCMV3-GP73-FLAG, pCMV3-GP73, pCMV3-HIF-1α-HA, pCMV3-VHL-FLAG and pCMV3-VHL-HA were purchased from Sinobiological (Beijing, China). The plasmids pcDNA3-GP73-CFP, pcDNA3-PHD-2-YFP, pcDNA3-HECTD1 and pcDNA3-His-Ub were purchased from OligoBio (Beijing, China). Truncated mutants of GP73 were constructed based on pCMV3-GP73-FLAG. The plasmids pLKO.1-GP73-Puro were constructed based on pLKO.1-Puro. Details on the shRNAs are shown in Supplementary Table 3. The siRNAs corresponding to human VEGFA, HIF-1α, GRB2 and HECTD1 were purchased from GenePharma (Shanghai, China). Details on the siRNAs are shown in Supplementary Table 3. Transfection of plasmids and siRNAs were performed using Lipofectamine 3000 (plasmids) and Lipofectamine RNAiMax (siRNAs) reagents (Thermo Fisher) following the manufacturer’s instructions.

**H&E and immunohistochemical staining**

Paraffin embedded sections of xenografts were manufactured in our previous study. H&E and immunohistochemical staining were performed as previously reported[1]. Antibodies used in immunohistochemical staining have been shown in Supplementary Table 2.

**MTS cell proliferation analysis**

HUVECs in 96-well-plate were cultured with culture medium incubated with indicated cells for 0, 24, 48, 72 h. Then, cell culture medium was replaced by serum-free culture medium with 10% MTS solution (G358C, Promega, Beijing, China) and incubated for extra 2 h. Results were quantified using a Multiskan^TM^ FC microplate reader (Thermo Fisher) at an absorbance of 490 nm.

**Transwell migration assay**

HUVECs (in upper chamber) were harvested 48 h after co-culturing with HCC cells (in lower chamber) or exosomes derived from indicated HCC cells (mixed with cell culture medium in upper chamber). Every chamber was seeded with 50,000 cells diluted in 100 μL serum-free DMEM. The bottom of the well was filled with 800 μL DMEM with 10% FBS. Cells were incubated in 5% CO_2_ at 37˚C for extra 48 h and fixed with methanol (>99.5%) for 5 m. After staining was conducted with 0.3% crystal violet, cells in the upper chamber were removed. Images were captured using an Olympus DP70 microscope in the bright field (Olympus Corporation, Tokyo, Japan).

**RNA isolation and Quantitative real-time PCR**

Total RNA was extracted using a TaKaRa MiniBEST Universal RNA Extraction Kit (9767, TaKaRa, Dalian, China). Genomic DNA was erased and cDNA first strands were synthesized using a PrimeScript™ RT reagent Kit with gDNA Eraser (RR047A, TaKaRa) according to the manufacturer’s instructions. Target mRNA expression was quantified via qRT-PCR using a CFX-96 Real-Time PCR system (Bio-Rad, Hercules, CA, USA). qRT-PCR was performed using TB Green^®^ Premix Ex Taq^™^ II (Tli RNaseH Plus, RR820A, TaKaRa) with the primers listed in Supplementary Table 4. The parameters of qRT-PCR were as specified in the manufacturer’s instructions. The mRNA level of β-actin was used as a reference.

**Extracellular VEGFA determination analysis**

The cell culture medium of HepG2 and MHCC-97H cells stably expressing vector, GP73, shV or GP73-specific shRNAs were replaced by DMEM with 10% FBS and cultured for an additional 24 h with in a Forma™ 3 incubator (4110, Thermo Fisher, Carlsbad, CA, USA) containing 2% O_2_. The cell culture medium was then collected; extracellular VEGFA was measured using a human VEGFA ELISA kit (ab119566, Abcam, Cambridge, MA, USA), following the manufacturer’s instructions.

**Immunofluorescence staining and confocal microscopy**

Immunofluorescence staining was performed as previously reported.(1) Antibodies used in immunofluorescence staining have been shown in Supplementary Table 2.

**Supplementary figures**

**
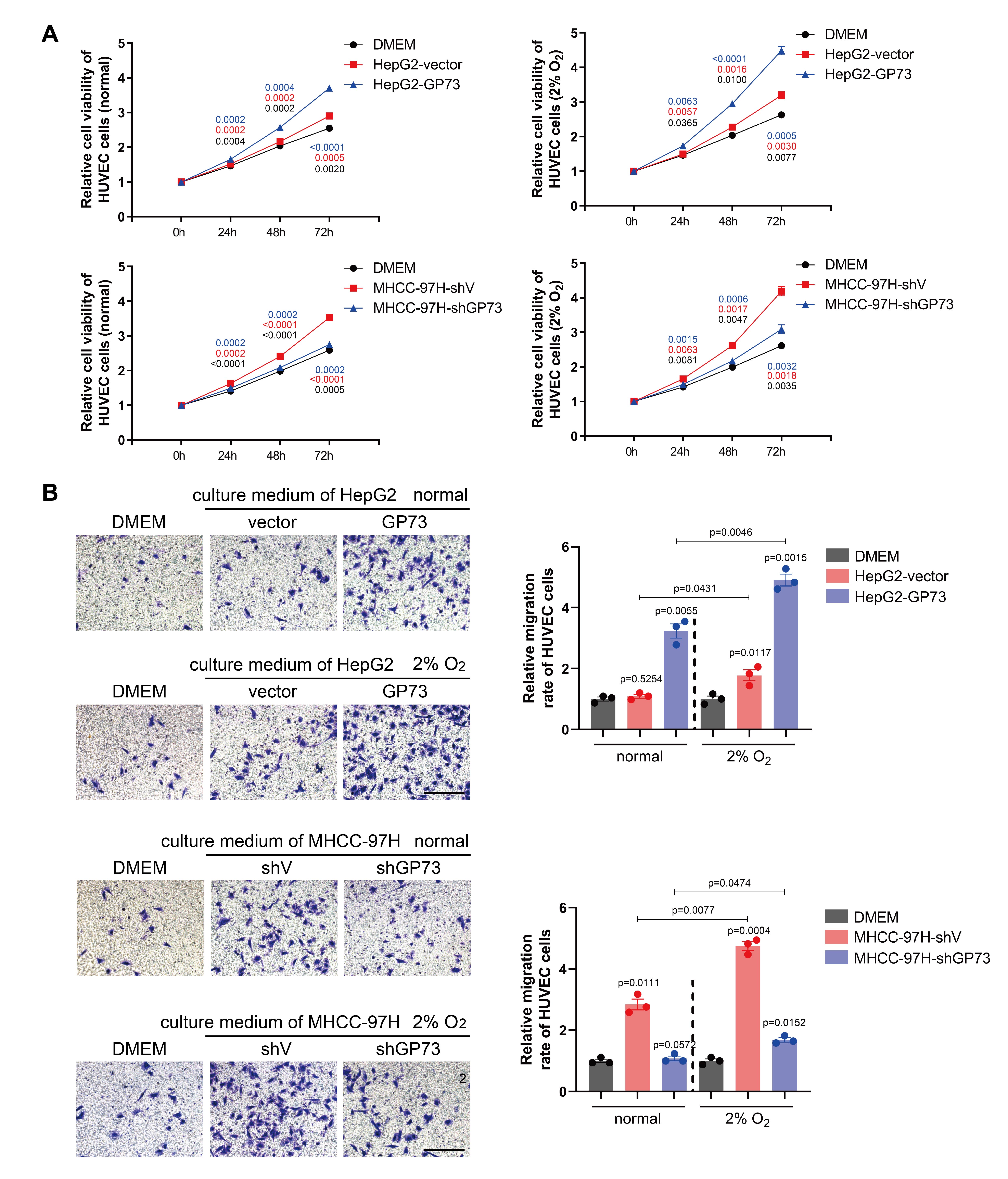
**

**Supplementary Fig. 1 GP73 facilitates angiogenesis in HCC.**

A. MTS assay of HUVECs treated with culture medium from indicated cells for 0, 24, 48 and 72 h (n=3).

B. Transwell cell migration assay of HUVECs (upper chamber) after co-culturing with culture medium from indicated cells (lower chamber) for 48 h (scale bar: 100 μm, n=3).

Data in A and B are presented as mean±s.e.m. A two-tailed Student’s *t*-test was used for statistical analysis.

**
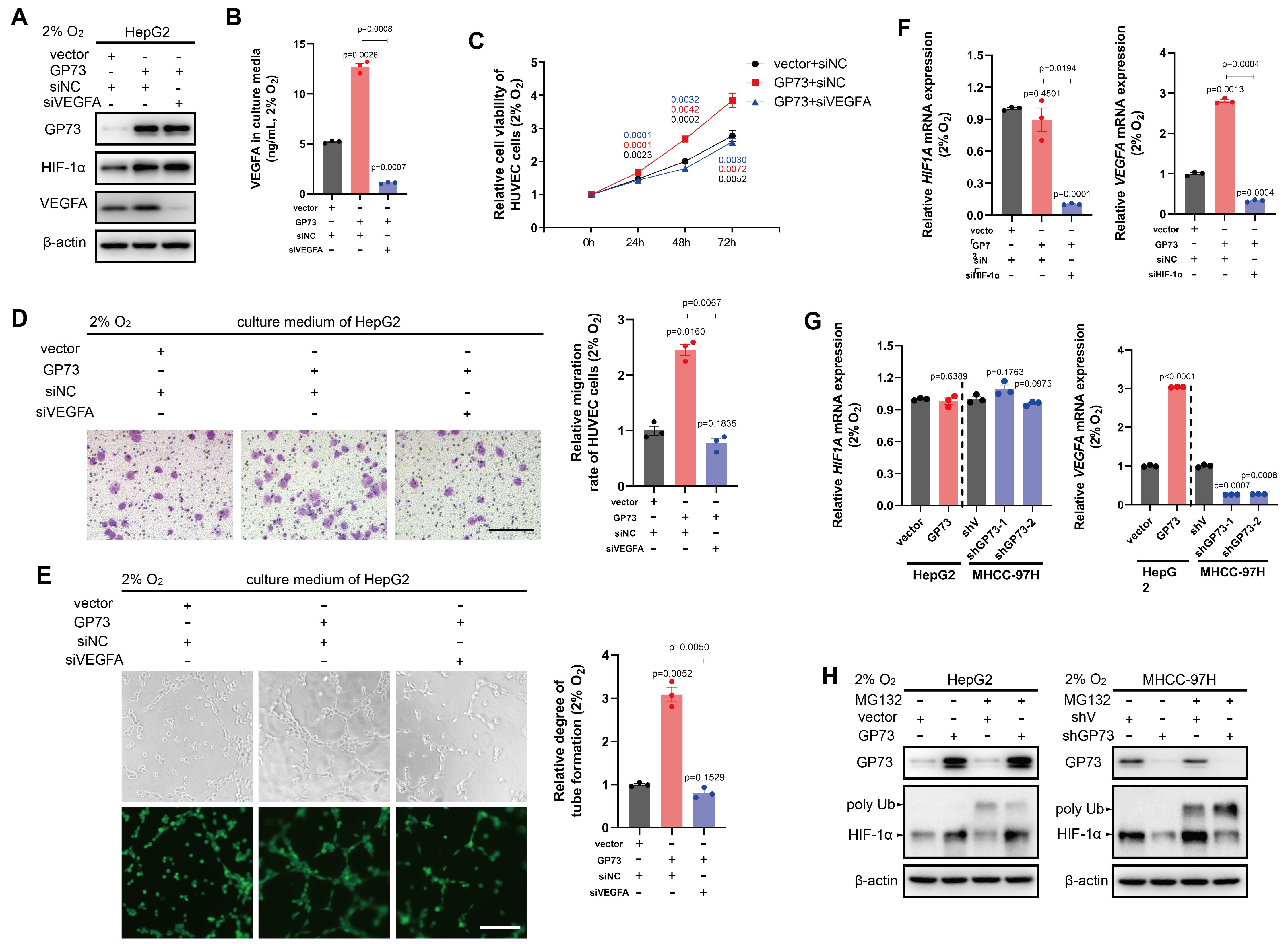
**

**Supplementary Fig. 2 GP73 stabilizes HIF-1α to promote VEGFA production in HCC cells.**

A. Immunoblotting analysis of HIF-1α and VEGFA in GP73-overexpressing HepG2 cells before and after 48 h treatment of VEGFA-specific siRNA (n=3).

B. The content of VEGFA in the cell culture medium from GP73-overexpressing HepG2 cells before and after 48 h treatment of VEGFA-specific siRNA (n=3).

C. Cell viability measured using MTS assay after HUVECs were treated with indicated cell culture medium for 0, 24, 48 and 72 h (n=3).

D. Transwell cell migration assay of HUVECs (upper chamber) after co-culturing with cell culture medium from HepG2 cells (lower chamber) for 48 h (scale bar: 100 μm, n=3).

E. Tube formation assay performed after HUVECs were treated with culture medium from HepG2 cells as indicated for 48 h (scale bar: 50 μm, n=3).

F. The mRNA levels of *HIF1A* and *VEGFA* in HepG2 cells as indicated were determined by qRT-PCR (n=3).

G. The mRNA levels of *HIF1A* and *VEGFA* in HepG2 and MHCC-97H cells as indicated were determined using qRT-PCR (n=3).

H. Ubiquitination level of endogenous HIF-1α in indicated cells was determined by immunoblotting analysis. Cells were treated with MG132 (2 μM, 12 h) before harvesting.

Data in B-G represent the mean ± s.e.m. A two-tailed Student’s *t*-test was used for statistical analysis.

**
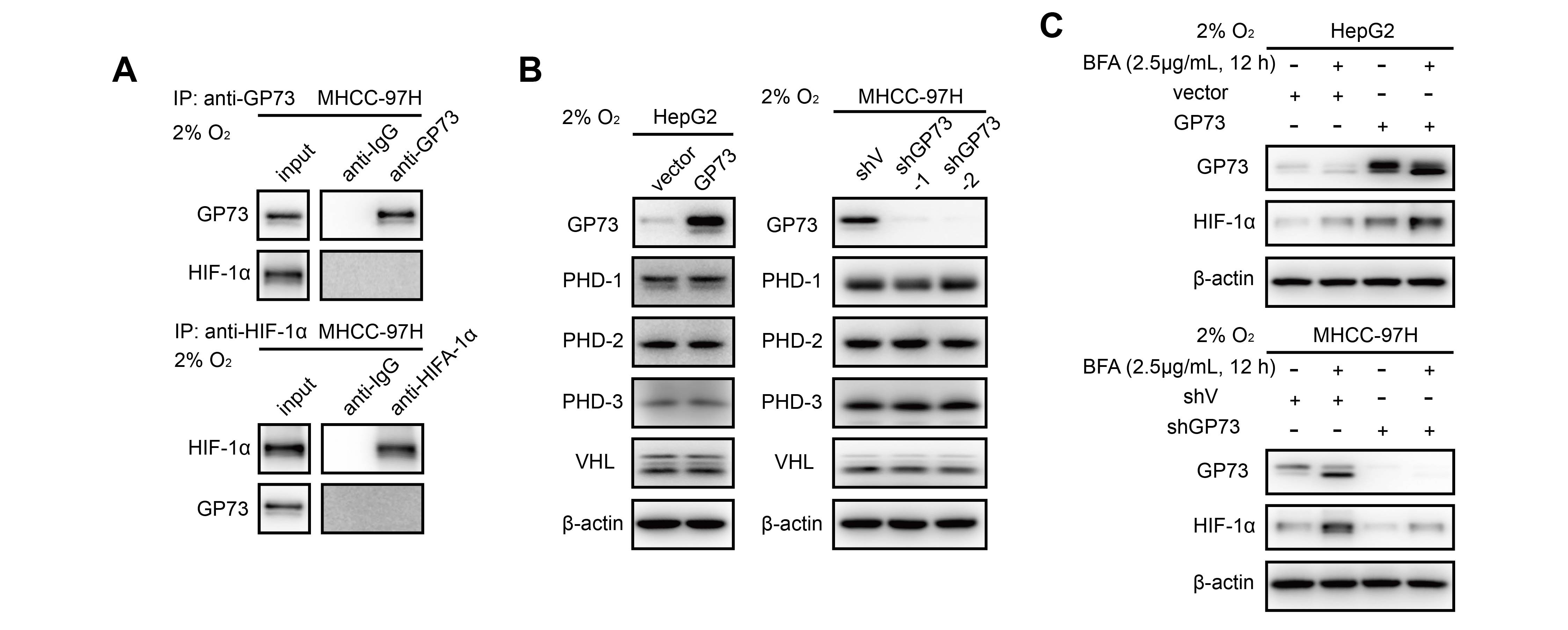
**

**Supplementary Fig. 3 GP73 interacts with PHD-2 to inhibit HIF-1α degradation in HCC cells.**

A. The interaction between GP73 and HIF-1α in MHCC-97H cells was determined using co-IP followed by immunoblotting analysis.

B. Immunoblotting analysis of HIF-1α degradation-related proteins in HepG2 and MHCC-97H cells.

C. Immunoblotting analysis of HIF-1α in HepG2 and MHCC-97H cells as indicated. Cells were treated with BFA (2.5 μg/mL) for 0 and 12 h.

**
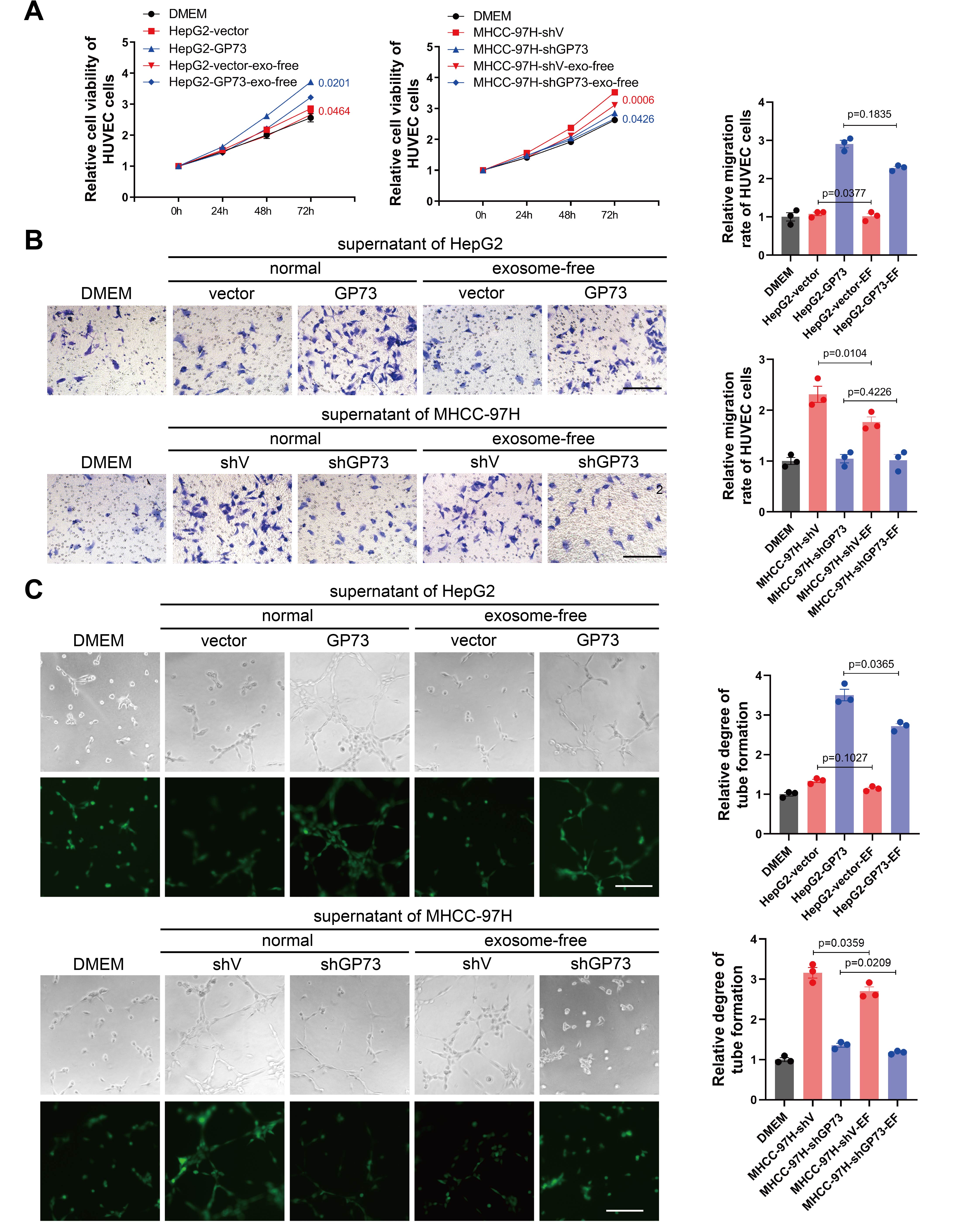
**

**Supplementary Fig. 4 Exosomes derived from HCC cells with GP73 upregulation potentially facilitates angiogenesis.**

A. MTS assay of HUVECs treated with cell culture medium from indicated cells for 0, 24, 48 and 72 h (n=3).

B. Transwell cell migration assay of HUVECs (upper chamber) after co-culturing with cell culture medium from indicated cells (lower chamber) for 48 h (scale bar: 100 μm, n=3).

C. Tube formation assay conducted after HUVECs were treated with cell culture medium from indicated cells for 48 h (scale bar: 50 μm, n=3).

Data in A-C represent the mean±s.e.m. A two-tailed Student’s *t*-test was used for statistical analysis.


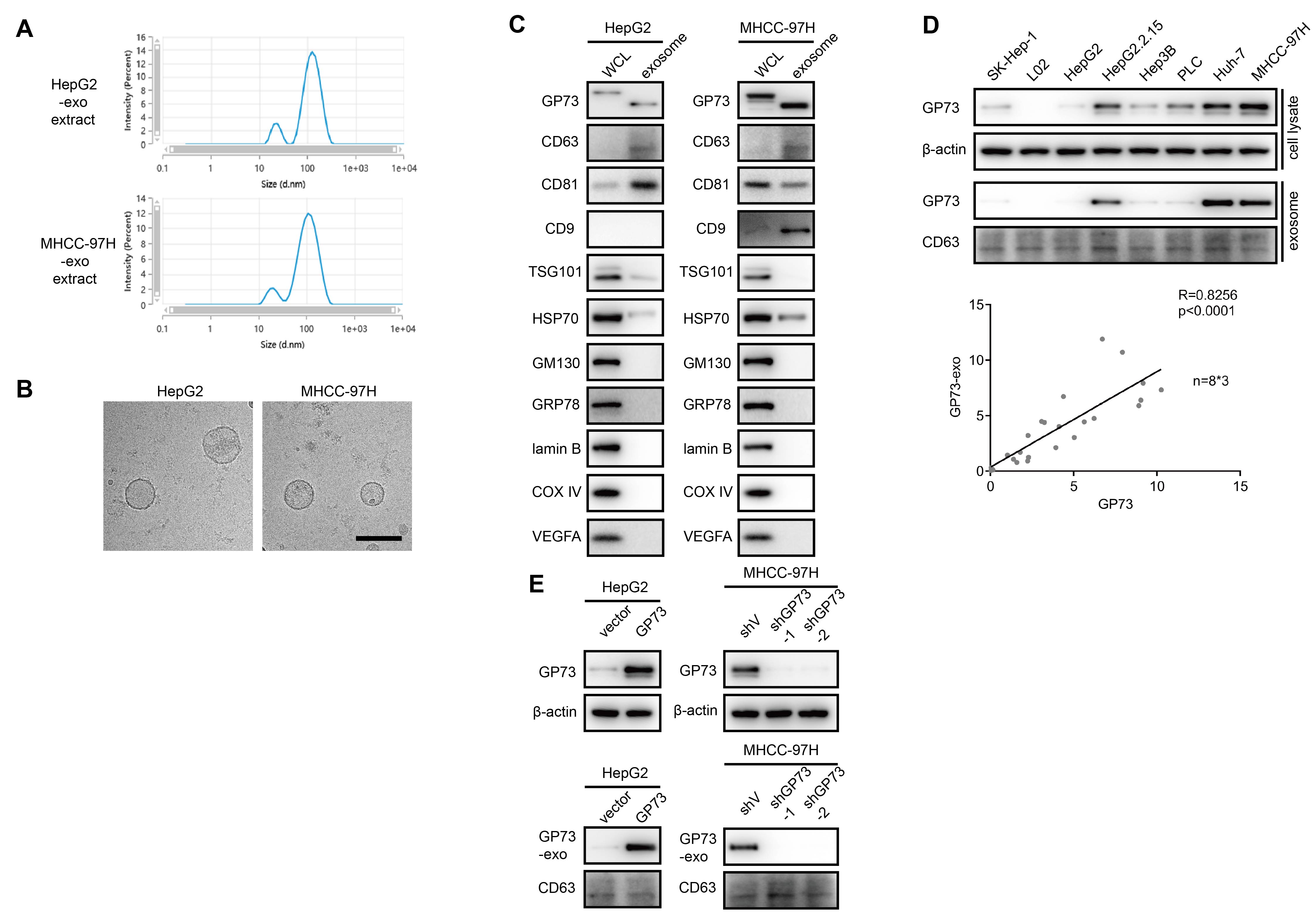


**Supplementary Fig. 5 Identification and quantification of GP73 in exosomes.**

A. Nanoparticle tracking analysis of HepG2 and MHCC-97H cells-derived exosomes.

B. Cryo-electron microscopy characterization of exosomes derived from HepG2 and MHCC-97H cells (scale bar: 200 nm).

C. Immunoblotting analysis of markers of intracellular vesicles, exosomes and subcellular components in whole cell lysates and exosomal extracts of HepG2 and MHCC-97H cells.

D. Immunoblotting analysis of intracellular and exosomal GP73 in whole cell lysates and exosomal extracts of normal liver and HCC cell lines. The correlation of intracellular and exosomal GP73 in indicated cell lines was represented using linear correlation.

E. Immunoblotting analysis of intracellular and exosomal GP73 in whole cell lysates and exosomal extracts of HepG2 and MHCC-97H cells (n=3).

Data in D were analyzed using a two-tailed Student’s *t*-test and spearman correlation analysis.


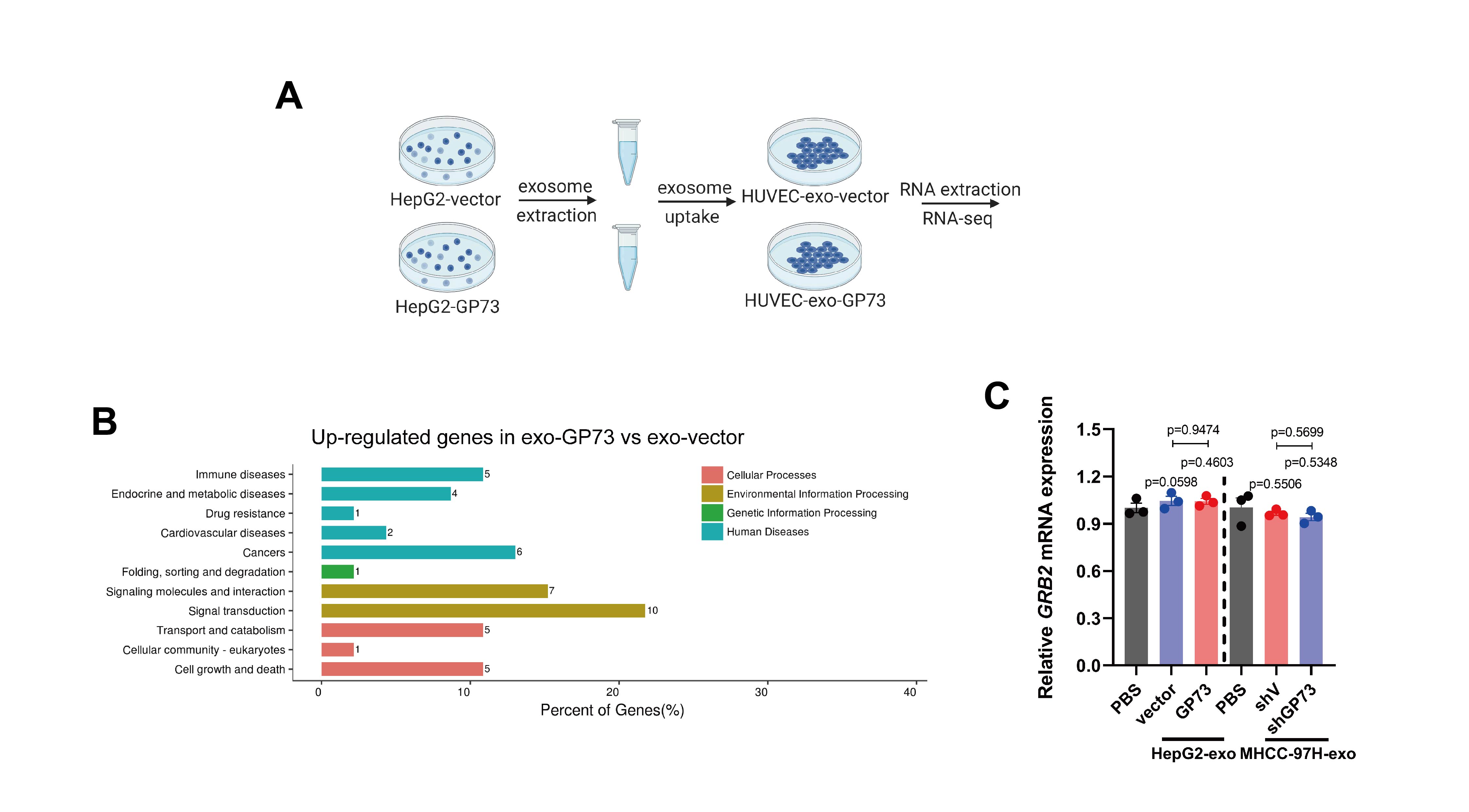


**Supplementary Fig. 6 Exosomes from GP73 upregulated HCC cells enhances Ras activation by stabilizing GRB2.**

A. Procedures of RNA-sequencing of HUVECs treated with exosomes derived from HepG2-vector and HepG2-GP73 cells for 48 h.

B. Gene Ontology (GO) analysis indicating that exosomes derived from GP73-upregulated-HCC cells activate cell growth and signal transduction-related signaling pathways.

C. The mRNA level of *GRB2* in HepG2 and MHCC-97H cells as indicated were determined using qRT-PCR (n=3).

Data in C represent the mean ± s.e.m. A two-tailed Student’s *t*-test was used for statistical analysis.


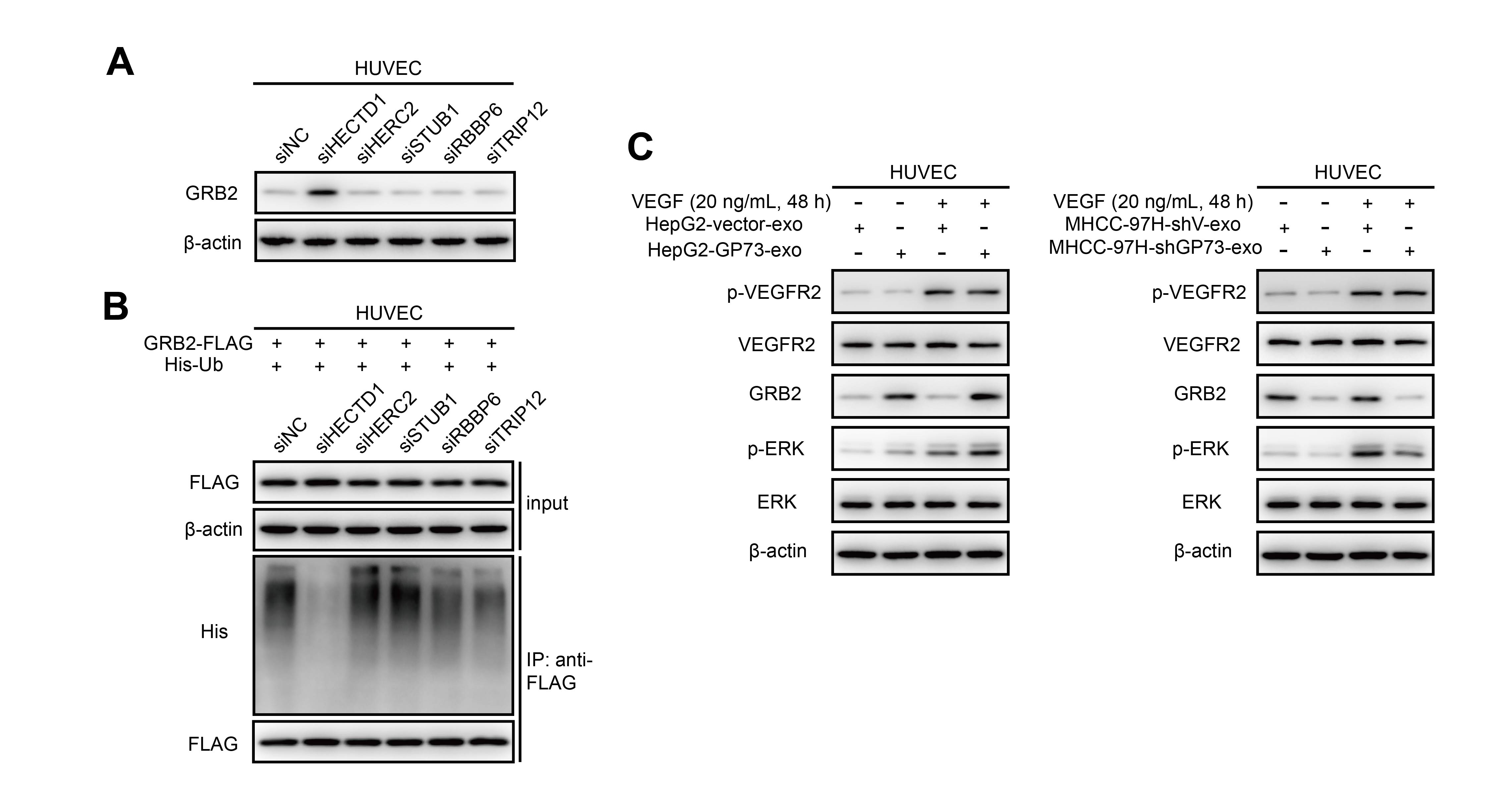


**Supplementary Fig. 7 Exosomal GP73 interacts with HECTD1 to inhibit GRB2 degradation and activates Ras/MAPK signaling in HUVECs.**

A. Immunoblotting analysis of GRB2 in HUVECs transfected with siRNAs targeting exosomal GP73-interacted E3 ubiquitin ligases for 48 h.

B. Ubiquitination level of GRB2-FLAG in HUVECs after transfection with siRNAs targeting exosomal GP73-interacted E3 ubiquitin ligases for 48 h, determined by immunoblotting analysis.

C. Immunoblotting analysis of ERK phosphorylation in HUVECs treated with exosomes derived from HepG2 and MHCC-97H cells.

**Supplementary tables**

**Table S1. Details of information of HCC patients.**

| Clinical valuable | Cohort (n=60) |
| --- | --- |
| Gender, n (%) |  |
| Male | 53 (88.3) |
| Female | 7 (11.7) |
| HBV infection, n (%) |  |
| Negative | 11 (18.3) |
| Positive | 49 (81.7) |
| Clinical stage, n (%) |  |
| I | 16 (26.7) |
| II | 23 (38.3) |
| III | 21 (35.0) |
| GP73 IHC score, median (IQR) |  |
| Tumor tissue | 5.0 (4.0, 8.0) |
| Normal tissue | 2.0 (1.0, 2.0) |
| CD31 IHC score, median (IQR) |  |
| Tumor tissue | 6.0 (3.0, 9.0) |
| Normal tissue | 1.0 (1.0, 2.0) |

The clinical profiles of patients were the same to the data of cohort 2 in our previous study[2]. In this study, we retested the levels of GP73 and CD31 using the identical HCC microarray.

**Table S2. Antibodies used for immunoblotting, immunofluorescence and immunohistochemical analyses.**

| Antibody | Company | Cat. NO. | Dilution/APPL |
| --- | --- | --- | --- |
| GOLPH2 (GP73) | Thermo Fisher | PA5-30622 | 1:3000 WB |
| HIF-1α | Cell Signaling Technology | 36169S | 1:3000 WB |
| VEGFA | Cell Signaling Technology | 50661S | 1:2000 WB |
| β-actin | EarthOX | E021020-01 | 1:10000 WB |
| 6*His-Tag | Proteintech | 66005-1-Ig | 1:1000 WB |
| PHD-1 | Proteintech | 12984-1-AP | 1:2000 WB |
| PHD-2 | Proteintech | 66589-1-Ig | 1:2000 WB |
| PHD-3 | Novus | NBP1-30440 | 1:1000 WB |
| VHL | Cell Signaling Technology | 68547S | 1:2000 WB |
| Hyd-HIF-1α (P564) | Cell Signaling Technology | 3434S | 1:1000 WB |
| FLAG-Tag | EarthOX | E022230-1 | 1:3000 WB |
| CD63 | Novus | NBP2-42252 | 1:200 WB |
| CD81 | Cell Signaling Technology | 52892S | 1:2000 WB |
| CD9 | Cell Signaling Technology | 13403S | 1:2000 WB |
| TSG101 | Abcam | Ab125011 | 1:2000 WB |
| HSP70 | Cell Signaling Technology | 4872S | 1:2000 WB |
| GM130 | Cell Signaling Technology | 12480T | 1:2000 WB |
| GRP78 (BiP) | Cell Signaling Technology | 3177T | 1:2000 WB |
| lamin B | Proteintech | 12987-1-AP | 1:5000 WB |
| COX IV | Cell Signaling Technology | 4850T | 1:5000 WB |
| GP73-exo | Raybiotech | 130-10311-100 | 1:1000 WB |
| Ras | Cell Signaling Technology | 4965S | 1:1000 WB |
| p-MEK1/2 (S217/221) | Cell Signaling Technology | 3958S | 1:2000 WB |
| MEK1/2 | Cell Signaling Technology | 9126S | 1:2000 WB |
| p-ERK (T202/Y204) | Cell Signaling Technology | 4370S | 1:2000 WB |
| ERK | Cell Signaling Technology | 4695S | 1:5000 WB |
| VEGFR2 | Cell Signaling Technology | 2479S | 1:1000 WB |
| p-VEGFR2 (Y1175) | Cell Signaling Technology | 3770S | 1:500 WB |
| GRB2 | Cell Signaling Technology | 3972S | 1:2000 WB |
| SOS1 | Cell Signaling Technology | 5890S | 1:1000 WB |
| HECTD1 | Proteintech | 20605-1-AP | 1:2000 WB |
| HA-Tag | Cell Signaling Technology | 3724S | 1:2000 WB |
| IPKine-M-R-IgG-LCS | Abbkine | A25022 | 1:10000 WB |
| IPKine-M-R-IgG-HCS | Abbkine | A25222 | 1:10000 WB |
| GOLM1 (GP73) | Abnova | H00051280-M04 | 1:200 IP |
| HIF-1α | Cell Signaling Technology | 36169S | 1:50 IP |
| VEGFR2 | Cell Signaling Technology | 2479S | 1:100 IP |
| SOS1 | Cell Signaling Technology | 5890S | 1:100 IP |
| Anti-FLAG M2 Gel | Sigma-Aldrich | A2220 | 15 μL/IP |
| Anti-HA Affinity Gel | Sigma-Aldrich | E6779 | 15 μL/IP |
| GP73-exo | Raybiotech | 130-10311-100 | 1:50 IP |
| GOLPH2 (GP73) | Thermo Fisher | PA5-30622 | 1:1000 IF |
| PHD-2 | Proteintech | 66589-1-Ig | 1:100 IF |
| GOLM1 (GP73) | Abnova | H00051280B01P | 1:400 IF |
| FLAG M2 | Sigma-Aldrich | F1804 | 1:1000 IF |
| HECTD1 | Proteintech | 20605-1-AP | 1:100 IF |
| GP73-exo | Raybiotech | 130-10311-100 | 1:100 IF |
| GOLPH2 (GP73) | Thermo Fisher | PA5-30622 | 1:2000 IHC |
| CD31 (Anti-mouse) | Cell Signaling Technology | 77699S | 1:100 IHC |
| CD31 (Anti-human) | Cell Signaling Technology | 3528S | 1:1000 IHC |

**Table S3. Sequences of shRNAs and siRNAs.**

| Targets | shRNA sequence/sense and antisense chains (5’-3’) |
| --- | --- |
| shGP73-1 | CGAATAGAAGAGGTCACCAAA |
| shGP73-2 | GAACAGTGTGAGGAGCGAATA |
| siGP73-1 | S: GAACAGUGUGAGGAGCGAATT |
|  | A: UUCGCUCCUCACACUGUUCTT |
| siGP73-2 | S: GUUGAGAAAGAGGAAACCATT |
|  | A: UGGUUUCCUCUUUCUCAACTT |
| siVEGFA-1 | S: AGGGCAGAAUCAUCACGAATT |
|  | A: UUCGUGAUGAUUCUGCCCUTT |
| siVEGFA-2 | S: GCGCAAGAAAUCCCGGUAUTT |
|  | A: AUACCGGGAUUUCUUGCGCTT |
| siHIF-1α-1 | S: GUGAUGAAAGAAUUACCGATT |
|  | A: UCGGUAAUUCUUUCAUCACTT |
| siHIF-1α-2 | S: CCGCUGGAGACACAAUCAUTT |
|  | A: AUGAUUGUGUCUCCAGCGGTT |
| siGRB2-1 | S: GAUCUACAUCUGUCUCCAGTT |
|  | A: CUGGAGACAGAUGUAGAUCTT |
| siGRB2-2 | S: CAGAUAUUCCUGCGGGACATT |
|  | A: UGUCCCGCAGGAAUAUCUGTT |
| siHECTD1-1 | S: GCACUUUCUUACCAGCCCUTT |
|  | A: AGGGCUGGUAAGAAAGUGCTT |
| siHECTD1-2 | S: GCCCUGAUAGUUCUGUUCGTT |
|  | A: CGAACAGAACUAUCAGGGCTT |
| siHERC2 | S: GACUGUAGCCAGAUUGAAATT |
|  | A: UUUCAAUCUGGCUACAGUCTT |
| siSTUB1 | S: CGUCUGUUCGUGGGCCGAATT |
|  | A: UUCGGCCCACGAACAGACGTT |
| siRBBP6 | S: CACCGAUUCAGGUAUUACATT |
|  | A: UGUAAUACCUGAAUCGGUGTT |
| siTRIP12 | S: GCACUUCAGCAUACUGAAUTT |
|  | AUUCAGUAUGCUGAAGUGCTT |
| siNC | S: GCGACGAUCUGCCUAAGAUTT |
|  | A: AUCUUAGGCAGAUCGUCGCTT |

**Table S4. Sequences of primers used in qRT-PCR.**

| Gene | Primer sequences (5’-3’) |
| --- | --- |
| *GOLM1* | F: CAGCGCTGATTTTGAGATGAC |
|  | R: ATGATCCGTGTCTGGAGGTC |
| *HIF1A* | F: GGCGCGAACGACAAGAAAAA |
|  | R: GGCTGTGTCGACTGAGGAAA |
| *GRB2* | F: AAGCTACTGCAGACGACGAG |
|  | R: GCCGCTGTTTGCTAAGCATT |
| *ACTB* | F: TTCCAGCCTTCCTTCCTGGG |
|  | R: TTGCGCTCAGGAGGAGCAAT |

**References**

[1] Liu Y, Zhou S, Shi J, Zhang X, Shentu L, Chen Z, et al. c-Myc transactivates GP73 and promotes metastasis of hepatocellular carcinoma cells through GP73-mediated MMP-7 trafficking in a mildly hypoxic microenvironment. *Oncogenesis* **2019**;8:58.

[2] Hu X, Yuan S, Zhou S, Sun T, Wang C, Ying S, et al. Golgi-protein 73 facilitates vimentin polymerization in hepatocellular carcinoma. *Int J Biol Sci* **2023**;19:3694-708.
